# Assessment of Physicochemical, Microbiological and Toxicological Hazards at an Illegal Landfill in Central Poland

**DOI:** 10.3390/ijerph19084826

**Published:** 2022-04-15

**Authors:** Justyna Szulc, Małgorzata Okrasa, Adriana Nowak, Joanna Nizioł, Tomasz Ruman, Sławomir Kuberski

**Affiliations:** 1 Department of Environmental Biotechnology, Lodz University of Technology, 90-530 Łódź, Poland; adriana.nowak@p.lodz.pl; 2 Department of Personal Protective Equipment, Central Institute for Labour Protection—National Research Institute, 90-133 Łódź, Poland; maokr@ciop.lodz.pl; 3 Faculty of Chemistry, Rzeszów University of Technology, 35-959 Rzeszów, Poland; jniziol@prz.edu.pl (J.N.); tomruman@prz.edu.pl (T.R.); 4 Faculty of Process and Environmental Engineering, Lodz University of Technology, 93-005 Łódź, Poland; slawomir.kuberski@p.lodz.pl

**Keywords:** illegal landfill, bioaerosol, harmful biological agents, metagenome analysis, toxicological analysis, UHPLC-Q-ToF-UHRMS analysis

## Abstract

This study aimed to assess the physicochemical, microbiological and toxicological hazards at an illegal landfill in central Poland. The research included the analysis of airborne dust (laser photometer), the number of microorganisms in the air, soil and leachate (culture method) and the microbial diversity in the landfill environment (high-throughput sequencing on the Illumina Miseq); the cytotoxicity (*PrestoBlue*) and genotoxicity (alkaline comet assay) of soil and leachate were tested. Moreover, an analysis of UHPLC-Q-ToF-UHRMS (ultra-high-performance liquid chromatography-quadrupole-time-of-flight ultrahigh-resolution mass spectrometry) was performed to determine the toxic compounds and microbial metabolites. The PM_1_ dust fraction constituted 99.89% and 99.99% of total dust and exceeded the threshold of 0.025 mg m^−3^ at the tested locations. In the air, the total number of bacteria was 9.33 × 10^1^–1.11 × 10^3^ CFU m^−3^, while fungi ranged from 1.17 × 10^2^ to 4.73 × 10^2^ CFU m^−3^. Psychrophilic bacteria were detected in the largest number in leachates (3.3 × 10^4^ to 2.69 × 10^6^ CFU mL^−1^) and in soil samples (8.53 × 10^5^ to 1.28 × 10^6^ CFU g^−1^). Bacteria belonging to Proteobacteria (42–64.7%), Bacteroidetes (4.2–23.7%), Actinobacteria (3.4–19.8%) and Firmicutes (0.7–6.3%) dominated. In the case of fungi, Basidiomycota (23.3–27.7%), Ascomycota (5.6–46.3%) and Mortierellomycota (3.1%) have the highest abundance. Bacteria (*Bacillus*, *Clostridium*, *Cellulosimicrobium*, *Escherichia*, *Pseudomonas*) and fungi (*Microascus*, *Chrysosporium*, *Candida*, *Malassezia*, *Aspergillus*, *Alternaria*, *Fusarium*, *Stachybotrys*, *Cladosporium*, *Didymella*) that are potentially hazardous to human health were detected in samples collected from the landfill. Tested leachates and soils were characterised by varied cyto/genotoxins. Common pesticides (carbamazepine, prometryn, terbutryn, permethrin, carbanilide, pyrethrin, carbaryl and prallethrin), quaternary ammonium compounds (benzalkonium chlorides), chemicals and/or polymer degradation products (melamine, triphenylphosphate, diphenylphtalate, insect repellent diethyltoluamide, and drugs (ketoprofen)) were found in soil and leachate samples. It has been proven that the tested landfill is the source of the emission of particulate matter; microorganisms (including potential pathogens) and cyto/genotoxic compounds.

## 1. Introduction

In Poland, almost 123 million tonnes of solid waste were generated in 2020, of which 10.7% was municipal solid waste (MSW) (13.1 million tonnes), which is 342 kg of this type of MSW per capita [1]. Despite the growing number of supporters of the circular economy, striving to minimise the amount of waste generated and the use of unavoidable waste as a recycling resource, the problem of vast amounts of MSW continues to be urgent. Following European Union legislation [2], proper waste management includes a five-step waste management hierarchy. First of all, waste should be prevented, reused, then recycled, other forms of recovery, and, finally, neutralised (e.g., by landfilling). Despite these guidelines, a large proportion of business waste is still landfilled. The amount of waste stored in Poland in 2020 amounted to 46 million tonnes and occupied a total area of almost 8 thousand hectares. MSW was stored in 271 dedicated landfills. At the same time, over 2000 existing illegal “wild” landfills with a total area of almost 2 km^2^ were inventoried [1]. A similar problem with illegal landfilling has been reported in Estonia, Italy and France. More facilities of this type are located in France, the Czech Republic, Croatia, Russia and Hungary. On the other hand, statistical data show that the largest number of illegal landfills in Europe is in Albania (9046), Slovakia (8334) and Romania (7174) [3]. Illegal landfill sites pose a potential hazard of severe pollution. Unlike the orderly municipal landfills, they are totally unprotected. Neither a geomembrane layer separates them from the substratum, waterproof sealing, nor a separate protection zone [4]. Illegal landfill sites arise most frequently in the vicinity of MSW management plants, wastelands, roadsides, byways, unfenced private plots (particularly on the city outskirts), in forests and by the water courses [5].

The literature data indicate that the problem of illegal MSW disposal is global [6,7,8]. The harmfulness of such objects seems obvious. It manifests itself mainly in the contamination of surface and ground water’s soils, the destruction of aesthetic and landscape values and the exclusion from the use of agricultural and forest areas used for MSW storage [9,10,11]. The adverse effects depend mainly on the type and quantity of MSW and the duration of its residence. Illegal landfill sites are a source of threat resulting from the migration of toxic substances from deposited MSW (or leachates) into the soil profile and due to the blowing and settling of dust from this place [5,12]. Illegal landfill sites are also the source of soil pollution with heavy metals [4]. Elevated concentrations of copper, nickel, zinc, chromium and mercury were registered in the soil in their vicinity [13,14]. Individual reports indicate that the spreading of pathogenic microorganisms carried by birds, rodents and other small animals, which seek food in such places, is also dangerous [5]. Among the numerous works on illegal landfill sites, it is difficult to find studies assessing the microbiological quality of air. Similarly, no comprehensive analyses would assess the content of harmful substances in soil, leachate and air in illegal landfills.

The research aimed to comprehensively assess the physicochemical, microbiological and toxicological hazards at an illegal landfill, including determining airborne dust, the number and types of microorganisms in the air, soil and leachate using classical methods of high-throughput sequencing on the Illumina Miseq platform. The cytotoxicity of soil samples and drainage leachate from the landfill against three human cell lines were tested. Moreover, analysis of a targeted and untargeted UHPLC-Q-ToF-UHRMS (ultra-high-performance liquid chromatography-quadrupole-time-of-flight ultrahigh-resolution mass spectrometry) was performed to determine the toxic compounds and microbial metabolites in soils and leachate samples from an illegal landfill site. The metagenomic analysis of air from illegal landfills has been performed in the presented study for the first time. Hitherto, metabolomic and toxicological studies for soil and leachate from a landfill environment have not been carried out so comprehensively.

## 2. Materials and Methods

### 2.1. Tested Landfill Sites

**Figure 1:**
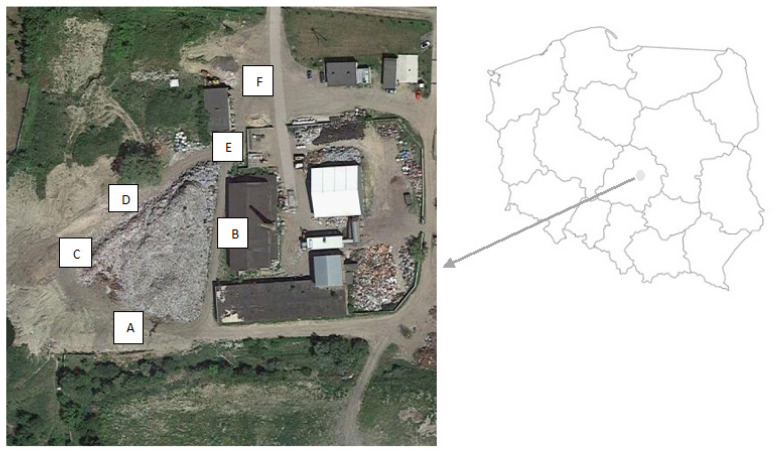
Tested landfill with an indication of the sampling sites.

**Figure 2:**
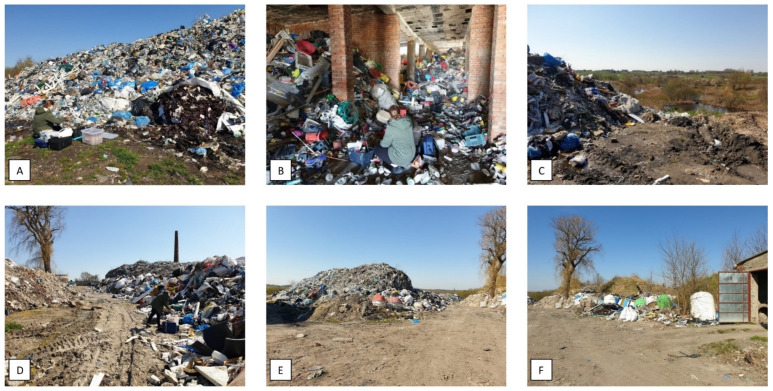
Sampling points at an illegal landfill; (**A**–**F**) described in Table 1.

Air, water and soil samples were collected at illegal landfills in a town 25 km away from Łódź (area 21.6 km^2^, population approx. 12,500; central Poland, Łódź Province)). The area is a former clay excavation site in which there has been a MSW landfill since the 1970s; it closed around 2015 and was recultivated. The area was used illegally to store ballast from the sorting plant. MSW has been stored without appropriate permits for about 10 years. The heap of MSW has a height of 218 m above sea level and a weight estimated at >5000 mg. The MSW is mixed with a predominance of plastics. The measurement sites are marked in Figure 1 and Figure 2 and described in Table 1.

### 2.2. Microclimate and Airborne Dust Concentration Analysis

A VelociCalc^®^ Multi-Function Velocity Meter 9545 (TSI, Shoreview, MN, USA) thermo-anemometer was used to establish the temperature, relative humidity and air-flow rate at the selected workstations. The particulate matter (PM) concentration was measured using a DustTrak™ DRX Aerosol Monitor 8533 portable laser photometer (TSI, USA). The detection range for particles ranging from 0.1 to 15 μm in size was between 0.001 and 150 mg m^−3^. The measurements were obtained in triplicates for each location at the height of 1.5 m from the ground level. The sampling rate was set at 3 L min^−1^ and the sampling interval at 5 s.

### 2.3. Sampling of Air, Leachate and Soil

Air samples for the determination of the number of microorganisms were collected using a MAS-100 Eco Air Sampler (Merck Life Science, Warsaw, Poland) according to the EN 13098 standard [15]. Three separate air samples of 50–100 L each, were collected at a height of 150 cm from the ground. Atmospheric air samples were also collected 250 m from the landfill and 10 km (external background). The air for the determination of biodiversity was collected using AirPort MD 8 (Sartorius, Goettingen, Germany) from place C. This location was crucial due to the potential spread of bioaerosol to nearby areas (presence of a stocked lake at the bottom of the escarpment). Sterile gelatine filters (80 mm, 0.3 µL Sartorius, Germany) were subjected to continuous air-flow of 8000 L and then sent for DNA extraction. Leachates and soils were collected in sterile 120 mL plastic containers. The leachates were collected directly into the containers, and the soils were sampled with a metal spatula from a depth of 5–20 cm.

### 2.4. Determination of Microorganism’s Number

The media summarised in Table 2 were used to determine the microbiological contamination of the air.

Moreover, the samples of soil (10 g) and leachate (10 mL) were suspended in 99 mL of sterile saline solution (0.85% NaCl). Dilutions were prepared from 10^−1^ to 10^−7^ in triplicates and then plated onto the above-mentioned media. In addition, TSC (Tryptose Sulfite Cycloserine Agar, Merck Life Science, Warsaw, Poland) was used to quantify the number of sulfate-reducing anaerobes.

All samples were incubated at the following temperature and time: 37 ± 2 °C, and 24–48 h (*Enterobacteriaceae*, mannitol-positive *Staphylococcus* sp.), 25 ± 2 °C and 5–7 days (actinomycetes, fungi, xerophilic fungi), 30 ± 2 °C and 48 h (bacteria, sulfate-reducing anaerobes, *Pseudomonas fluorescens*).

Following incubation, the colonies were counted. The results were expressed in CFU m^−3^ (air) and CFU g^−1^ (soil) CFU mL^−1^ (leachate). The arithmetic mean of three independent repetitions was reported as a result.

### 2.5. Determination of Biodiversity

A modified method based on Genomic Mini AX Bacteria + set (A&A Biotechnology, Gdańsk, Poland) was used to extract genomic DNA. A FastPrep-24 device was used to perform additional mechanical lysis of the samples. Additional DNA treatment was performed with the Anti-Inhibitor Kit (A&A Biotechnology, Gdańsk, Poland) post-extraction. The Real-Time PCR was used to confirm the presence of bacterial DNA in the tested samples. The Real-Time PCR reaction was carried out in Mx3000P thermocycler (Stratagene, La Jolla, CA, USA), using SYBR Green dye as a fluorochrome.

The extracted DNA concentration was between 2 and 30 µg mL^−1^. Universal primers amplifying the 16S rRNA bacterial gene’s fragment and fungal ITS regions were used in the reaction [16,17]. The DNA eluates had been checked for their quality and quantity prior to library preparation. The libraries of V3–V4 and ITS amplicons were prepared according to the guidelines of 16S Metagenomic Sequencing Library Preparation Part #15044223 Rev. B. The Herculase II Fusion DNA Polymerase Nextera XT Index Kit V2 was used for the two-step PCR.

The libraries were performed and quality-checked according to the Illumina qPCR Quantification Protocol Guide and the sequencing Macrogen (Seoul, Korea). Then, the sequencing was performed using the paired-end technology on the Illumina MiSeq (2 × 300 bp) platform (Macrogen; Seoul, Korea).

Qualitative and quantitative taxonomic identification was carried out as described in [18], using CLC Genomic Workbench v. 12 (Qiagen, Hilden, Germany) + Microbial Genomics Module Plugin v. 4.1 (Qiagen).

### 2.6. Toxicological Tests

#### 2.6.1. Cell Cultures

Caco-2 cells (human colon adenocarcinoma) were cultured in high-glucose DMEM (Dulbecco’s Modified Eagle’s Medium, Merck Life Science, Warsaw, Poland), A-549 cells (human lung alveolar adenocarcinoma) in DMEM:Ham’s F12 (1:1, *v*/*v*), Hep-G2 (human hepatocellular carcinoma) in Ham’s F12 (Merck Life Science, Warsaw, Poland), while IEC-6 (rat normal small intestine) in low-glucose DMEM:RPMI 1640 (1:1, *v*/*v*, Merck Life Science, Warsaw, Poland), with the addition of 5% (A-549, Hep-G2, IEC-6) or 10% (Caco-2) FBS (fetal bovine serum), 2 (A-549, Hep-G2, IEC-6) or 4 mM (Caco-2) GlutaMAX^TM^ (Thermo Fisher Scientific, Waltham, MA, USA), 25 mM HEPES (Merck Life Science, Warsaw, Poland), 100 µg/mL streptomycin/100 IU/mL penicillin (Merck Life Science, Warsaw, Poland) and 0.1 U/mL insulin (IEC-6). Caco-2, A-549 and Hep-G2 were purchased from Cell Line Service GmbH (Eppelheim, Germany), while IEC-6 from DSMZ German Collection of Microorganisms and Cell Cultures GmbH (Germany). The cells were then incubated at 37 °C with 5% CO_2_ in the humidified atmosphere for 7–10 days to reach 80% confluence. Two to three times per week, the cells were washed with 0.1 M PBS (pH 7.2), and the medium was renewed. TrypLE^TM^ Express (Thermo Fisher Scientific, Waltham, MA, USA) was used to detach the cells from the culture (37 °C, 6–12 min). Then the cells were centrifuged (307× *g*, 5 min) and decanted. Then, the pellet was re-suspended in a fresh culture medium. The cells were ready to use after performing a cell count by hemacytometer and determining cell viability by trypan blue test (it must have been at least 90%).

#### 2.6.2. Preparation of Samples

Soil samples (1.0 g) were suspended in 10 mL of the appropriate cell culture-ready medium, mixed and then extracted for 40 min (160 r.p.m.) at ambient temperature. The primary soil concentration in each extract was 100 mg/mL. The pH of each extract was adjusted to neutral (pH 7.0 ± 0.2) to eliminate the influence of this parameter on the toxicity. Sterile 0.22 µm syringe filters (Membrane Solutions, Kent, WA, USA) were used to filter each extract twice. The leachate samples were only filtered through sterile 0.22 µm syringe filters. A series of dilutions were prepared.

#### 2.6.3. Cytotoxicity and IC_50_ Determination

The cytotoxicity of the water-soluble fractions of the soil samples and the leachate samples was assessed with *PrestoBlue*. A total of 5000 (Caco-2, Hep-G2, IEC-6) or 1000 (A-549) cells/well were placed in 96-well black flat-bottom plates and incubated for 24 h at 37 °C in 5% CO_2_. The next day, the medium was removed from the cells, and then dilutions of leachate samples and soil extracts were added. The final concentrations of the analysed soil extracts were (mg/mL): 3.13; 6.25; 12.5; 25; 50; 100, while for the leachate samples (mg/mL): 31.3; 62.5; 125; 250; 500. Cells in the culture medium were negative control. The samples were exposed for 72 h at 37 °C in an atmosphere of 5% CO_2_. After this, the samples were aspirated, and *PrestoBlue* (10% solution in PBS) was added to each well and incubated for a further 2 h at 37 °C in 5% CO_2_. A microplate reader (TriStar2 LB 942, Berthold Technologies GmbH & Co. KG, Bad Wildbad, Germany) was used to measure fluorescence (λ_ex_ 560 nm; λ_em_ 590 nm). IC_50_ values were estimated from the resulting curves.

#### 2.6.4. Single Cell Gel Electrophoresis Assay

The tested samples/dilutions were introduced into Eppendorf tubes along with A-549 cells in the amount of 1 × 10^5^ cells/sample (the final volume was 1 mL). The negative control contained cells suspended in the culture medium. Cells incubated with 25 µM H_2_O_2_ were a positive control. The samples were incubated for 60 min at 37 °C, centrifuged (15 min, 4 °C, 182× *g*), decanted and LMP (*Low Melting Point*) agarose was added at 37 °C. The suspension was spotted on warm NMP (*Normal Melting Point*) double-layered slides and covered with coverslips (hot plate ZF6 Premiere Slide Warmer). The samples were placed on a Chilling Plate for Comet Assay Slides (Cleaver Scientific) and allowed to solidify. Then, alkaline lysis was performed with the buffer (2.5 M NaCl, 1% Triton X-100, 100 mM EDTA, 10 mM Tris, pH 10) and incubated (60 min, 4 °C). The lysis buffer was decanted, and then the slides were flooded with the unwinding buffer (300 mM NaOH, 1 mM EDTA) (20 min, 4 °C); next, they were placed in an electrophoresis apparatus (CSL-COM20, Cleaver Scientific). The electrophoresis was performed in an electrophoretic buffer (300 mM NaOH, 1 mM EDTA, pH > 13) for 20 min at a voltage of 21 V and a current of 29 mA. The slides were neutralised in leachate, allowed to dry, and then were stained for 60 min at 4 °C with DAPI (1 µg/mL); then, comet analysis was performed under a fluorescence microscope (Nikon) at a magnification of 200× equipped with a camera (Nikon Digital Sight DS-U3) and with Lucia Comet v.7.0 software (Laboratory Imaging, Prague, Czech Republic). In each trial, 50 randomly selected comets were analysed based on the parameter determining the percentage of DNA in the comet’s “tail”. Results are presented as mean ± S.E.M.

### 2.7. UHPLC-Q-ToF-UHRMS Analysis

#### 2.7.1. Preparation of Samples

Soil samples (2.0 g) were suspended in 2 mL LC-MS-grade methanol (Sigma-Aldrich, Poznań, Poland), vortexed for 5 min and left for extraction for 1 h at room temperature. The resulting suspensions were filtered with the use of syringe filters (0.22 µm pores). The leachate samples were only filtered through sterile 0.22 µm syringe filters. The soil and leachate samples were pipetted into standard HPLC vials and inserted into a Bruker Elute autosampler. The thermostated chamber of the autosampler was set at 5 °C.

#### 2.7.2. Instrumentation

UHPLC-Q-ToF-UHRMS analysis was performed on a Bruker Elute UHPLC system operated by Hystar 3.3 software and a Bruker Impact II (Bruker Daltonik GmbH, Bremen, Germeny) mass spectrometer of ESI QToF-MS type. The column for the Elute system was Bruker Intensity Solo with C18 silica modification, 1.8 μm particles, 100 × 2.1 mm (length × diameter). The UHPLC column was thermostated and held at 40 °C. The first mobile phase was water:methanol 99:1 (*v*/*v*) with 5 mM NH_4_HCOO and 0.01% HCOOH. Phase B was methanol 5 mM NH_4_HCOO and 0.01% HCOOH. The injection volume was 5 μL. For Targetscreener bbCID measurements the B percentage was: 18.3% (1 min), 50% (2.5 min), 99.9% (14–16 min), 4% (16.1–20 min). The solvent flow was 0.2 mLmin^−1^ from 0 to 1 min, gradually changing from 0.2 to 0.223 mL min^−1^ from 1 to 2.5 min and from 0.223 to 0.400 μLmin^−1^ from 2.5 to 14 min; after this, the flow was 0.4 to 0.48 mL min^−1^ from 14 to 19 min and back to 0.2 mL min^−1^ from 19.1 to 20 min. The UHPLC separations for the AutoMSMS experiments were conducted similarly, flows and B phase percentages were identical as above, with the exception that 99.9% of B was held up to 25 min at 0.48 mL min^−1^ and 4% B was from 26.1 to 30 min. Internal calibration was based on ions from 10 mM sodium formate pumped into the ESI ion source with a syringe pump at an infusion flow rate of 0.12 mL h^−1^ (solvent: water: isopropanol 1:1 *v*/*v*). The calibration was performed automatically in Metaboscape using a high precision calibration (HPC) mode. The analyses in positive bbCID mode were made with the following parameters: pre-pulse storage time: 8 μs; transfer time: 60 μs *m*/*z*: 50–1000; capillary voltage: 4 kV; nebuliser: 4 bar; dry gas: 8 L min^−1^; drying gas temperature: 200 °C; hexapole voltage: 30 Vpp; funnel 1: 300 Vpp; funnel 2: 300 Vpp. In autoMSMS mode, the *m*/*z* range was 50–1500, and the CID (Collision-Induced Dissociation) energy value was 30 eV. The CID settings were: absolute area threshold: 5000 cts; active exclusion 2; isolation window: for *m*/*z* = 100-4, 300-5, 500-6, 1000-8. Targeted identification was made in TASQ (ver. 2021b) with the use of built-in libraries containing spectral details and additionally retention times. The untargeted annotations were performed in Metaboscape (ver. 2021b) with a criterion of mass deviation (Δ *m*/*z*) under 2 ppm and a mSigma value under 15. MSMS spectra were automatically matched against MSMS libraries: Bruker HMDB 2.0 library, MoNA library [19] and NIST ver. 2020 MSMS library [20].

### 2.8. Statistical Analysis

#### 2.8.1. Microclimate, Airborne Dust Concentration and Microbial Contamination Analysis

Statistical analysis was carried out with Statistica 13.1 (Statsoft, Tulsa, OK, USA). Descriptive statistics were calculated for all variables of interest. The numbers of microorganisms in the air, soil, and leachate were compared between the tested samples (ANOVA, α = 0.05). Tukey’s post hoc procedure was used to compare the means (α = 0.05) in case a statistical difference was detected (*p* < 0.05).

#### 2.8.2. Toxicological Analysis

A two-way analysis of variance (ANOVA) was conducted using OriginPro 6.1 (Northampton, MA, USA) software to evaluate the experimental data for genotoxicity testing. The significant differences between the means were compared using Scheffe’s multiple comparison test, and they were regarded as significant at *p* < 0.05.

#### 2.8.3. Metabolite and Pathway Analysis

All metabolites were analysed using the advanced MetaboAnalyst 5.0 online software to perform multivariate statistical analysis [21]. Metabolite profiles of leachate and soil samples from UHPLC-HRMS analysis were subjected to unsupervised Principal Component Analysis (PCA). The separation between two groups of leachates (2, 3 vs. 1, 4, 5) and two groups of soils (2, 4, 5 vs. 1, 3) was examined using Orthogonal Partial Least Squares Discriminant Analysis (OPLS-DA). The overall quality of the OPLS-DA models was assessed by examining the goodness of fit (R^2^Y) and the predictive ability of the models (Q^2^). Potential features for group separation of leachate samples were subsequently identified by S-plot loading analysis of corresponding OPLS-DA models and based on the significance criterion of |p(corr)| > 0.5 and |p| > 0.05. The data were analysed using an independent *t*-test and fold-change analysis. *p*-values and false discovery rates (FDR; q-value) less than 0.05 and |Log_2_ FC| > 1 were considered statistically significant.

All 2666 (for leachate) and 2413 (soil samples) identified compounds by LC-MS were analysed using MetaboAnalyst 5.0 software to identify the most relevant pathways. However, targeted pathway analysis was performed for 471 (leachate) and 413 (soil) compounds that were successfully well-annotated by HMDB or KEGG ID. Metabolic pathways with values > 0.1 were considered to be significant. Pathway enrichment analysis was performed based on the main class compounds.

## 3. Results and Discussion

### 3.1. Microclimate and Airborne Dust Concentration

**Figure 3:**
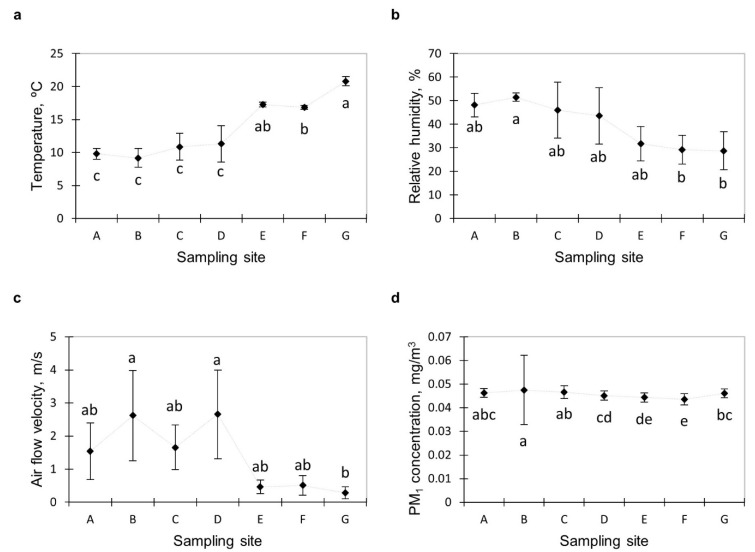
Microclimate and airborne dust concentration parameters in tested landfill site; (**a**) temperature, (**b**) relative humidity, (**c**) air-flow velocity, (**d**) concentration of dust. Statistically different samples within the same microorganism group were marked with different letters (Tukey’s test at *p* = 0.05).

The microclimate parameters of the illegal dumping are summarised in Figure 3. At location B, the lowest temperature (9.2 °C) and the highest relative humidity (51.5%) and air-flow rate (2.62 m s^−1^) were observed. The highest temperature (20.8 °C) and the lowest relative humidity (28.7%) and air-flow rate (0.28 m s^−1^) were observed for location G, which was an external control. The microclimate conditions in different locations were generally similar (for locations A–D, no statistical differences in all tested parameters were detected, and for E, only the temperature was different from locations A–D). Statistically significant differences were detected between tested locations and internal and external control (temperature between A–D vs. F and G, relative humidity for B vs. F and G, and air-flow rate between B and D vs. F and G). The diversified values of the microclimate parameters in the tested locations and both control sampling sites suggest that microorganism development conditions might differ in those locations.

The PM_1_ size fraction (Figure 3d), i.e., particles with dimensions below 1 µm, constituted almost all the measured dust at the tested locations. Its share in the total quantity of the measured dust was between 99.89% and 99.99%. The PM_1_ dust concentration at tested locations was similar, but statistically significant differences occurred either way. The highest values were observed at location B (0.0475 mg m^−3^) and the lowest ones at location F, which constituted an internal control (0.0436 mg m^−3^). Such results can be associated with air-flow velocities that were much higher for location B than for location F. Exposure to air pollution can cause significant harm to humans. To improve air quality, the European Union has developed a set of health-based standards and objectives for various air pollutants [22]. According to the Directive’s requirements, the annual average concentration of dust with dimensions below 2.5 µm (i.e., the fraction containing the PM_1_ fraction) should not exceed 0.025 mg m^−3^. It means that the measured PM_1_ concentration was almost twice as high as the environmental threshold independent of the sampling site.

### 3.2. Number of Microorganisms in the Air, Leachate and Soil

**Figure 4:**
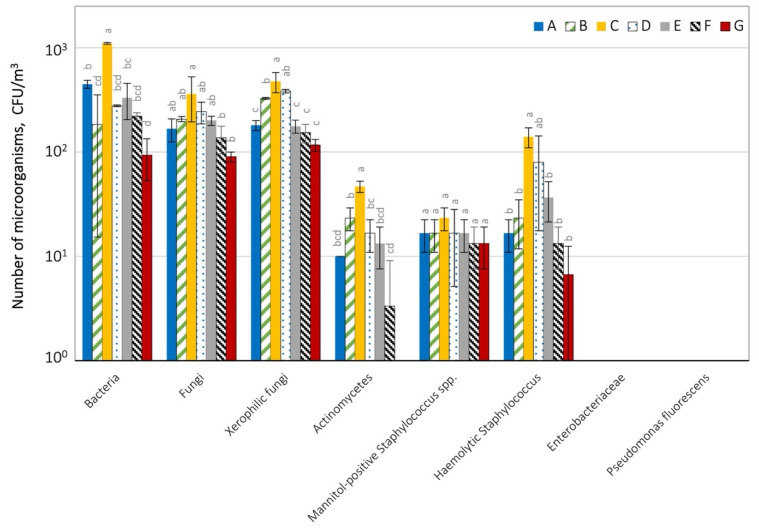
Microbiological air contamination at the tested landfill; A–G air sampling sites; statistically different samples within the same microorganism group were marked with different letters (Tukey’s test at *p* = 0.05).

The number of microorganisms in the air at the landfill is shown in Figure 4 and Table S1 (Supplementary Materials).

In the air in the tested landfill, the total number of bacteria ranged from 9.33 × 10^1^ (G, external control) to 1.11 × 10^3^ CFU m^−3^ (place C). The number of bacteria at site C was statistically greater (*p* = 0.05) than in the other investigated locations. The number of fungi ranged from 1.17 × 10^2^ (F, internal control) to 4.73 × 10^2^ CFU m^−3^ (C).

**Figure 5:**
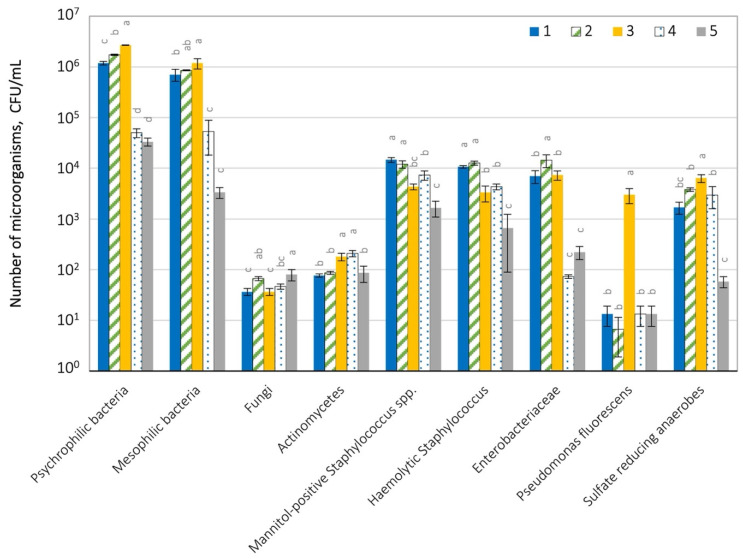
Microbiological contamination of leachate in the tested landfill; 1–5 leachate sample numbers; statistically different samples within the same microorganism group were marked with different letters (Tukey’s test at *p* = 0.05).

The number of xerophilic fungi was similar, from 1.17 × 10^2^ to 4.47 × 10^2^ CFU m^−3^. A lower number was found in the case of mannitol-positive staphylococci (1.33 × 10^1^ to 2.33 × 10^1^ CFU m^−3^) and hemolytic bacteria (6.67 × 10^0^ to 1.40 × 10^2^ CFU m^−3^). Actinomycetes were the least numerous in the tested air samples (33.3 × 10^0^ and 4.67 × 10^1^ CFU m^−3^). However, their statistically significant dominance (*p* = 0.05) at place C was observed compared to the other examined places. No presence of *Enterobacteriaceae* bacteria, which could be evidence of faecal contamination of the air, or *Pseudomonas fluorescens* bacteria were recorded at the examined locations. Regardless of the type of microorganisms, the greatest number were recorded at site C (pile of MSW on the hill on the west side) and the lowest in the samples from external control (site G) (Figure 5, Table S1).

The assessment of the microbiological contamination of the leachates from the dumping area is presented in Figure 5 and Table S2 (Supplementary Materials).

The predominance of psychrophilic bacteria was found in the tested samples, which were detected in the number from 3.3 × 10^4^ (sample no. 5) to 2.69 × 10^6^ CFU mL^−1^ (no. 3). The number of bacteria at location 3 was statistically greater (*p* = 0.05) than in the other investigated locations. Mesophilic bacteria were also abundant (from 3.37 × 10^3^ to 1.18 × 10^6^ CFU mL^−1^). A smaller number was found for mannitol-positive staphylococci (6.67 × 10^2^–1.27 × 10^4^ CFU mL^−1^), hemolytic bacteria (1.67 × 10^3^–1.47 × 10^4^ CFU mL^−1^), sulphate-reducing anaerobic bacteria (5.83 × 10^1^–6.37 × 10^3^ CFU mL^−1^) in tested leachate samples.

*Pseudomonas fluorescens* bacteria (6.67 × 10^0^–3.0 × 10^3^ CFU mL^−1^) and bacteria from the *Enterobacteriaceae* family (7.33 × 10^1^–1.43 × 10^3^ CFU mL^−1^) were also present in the leachates. Low contamination was found for fungi (3.67 × 10^1^–8.0 × 10^1^ CFU mL^−1^) and actinomycetes (7.67 × 10^1^–2.1 × 10^2^ CFU mL^−1^). It is noteworthy that the contamination of *Enterobacteriaceae*, *Pseudomonas fluorescens* bacteria and sulphate-reducing anaerobic were statistically significantly higher (*p* = 0.05) in the case of no. 3 (collected from location A) compared to the other studied samples.

**Figure 6:**
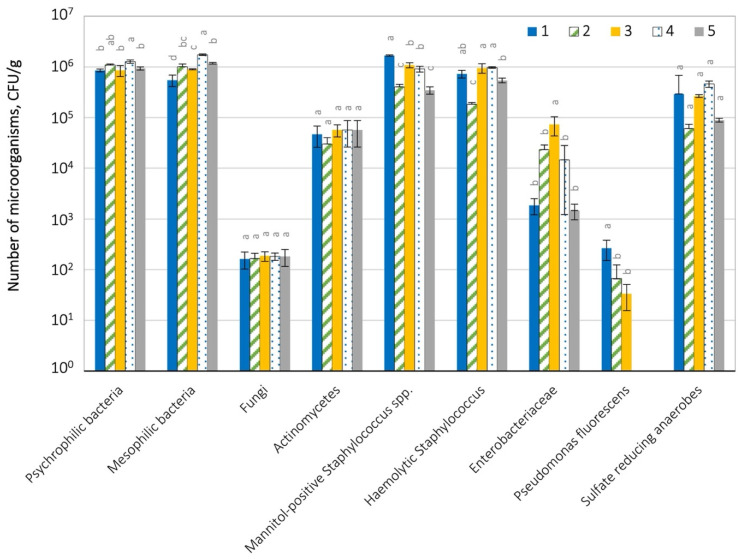
Microbiological contamination of soil in the tested landfill; 1–5 leachate sample numbers; statistically different samples within the same microorganism group were marked with different letters (Tukey’s test at *p* = 0.05).

The assessment of microbial contamination of the soil collected at the illegal landfill is presented in Figure 6 and Table S3 (Supplementary Materials).

Psychrophilic bacteria were detected in the largest number in these samples-from 8.53 × 10^5^ to 1.28 × 10^6^ CFU g^−1^ and mesophiles-from 5.49 × 10^5^ to 1.74 × 10^6^ CFU g^−1^. A high number of hemolytic bacteria (3.0 × 10^4^–9.7 × 10^5^ CFU g^−1^), mannitol-positive staphylococci (3.47 × 10^5–^1.68 × 10^6^ CFU g^−1^) and anaerobic bacteria (6.03 × 10^4^–4.59 × 10^6^ CFU g^−1^) were also observed. Moreover, the presence of Actinomycetes ranging from 3.0 × 10^4^ to 5.67 × 10^4^ CFU g^−1^ was found. *Enterobacteriaceae* bacteria were also detected at the level of 1.47 × 10^3^–7.32 × 10^4^ CFU g^−1^), and *Pseudomonas fluorescens* bacteria in 3 out of 5 examined samples (3.3 × 10^1^–2.67 × 10^2^ CFU g^−1^). In the soil samples, a low number of fungi was detected, ranging from 1.67 × 10^2^ to 1.87 × 10^2^ CFU g^−1^. The tested soil samples were slightly differentiated in terms of the number of microorganisms. No statistically significant differences (*p* = 0.05) were found in the number of fungi, Actinomycetes and sulphate-reducing anaerobes between individual samples.

Many studies indicate that landfills are a significant epidemiological threat due to the possibility of the presence and multiplication of pathogenic bacteria in MSW. This is especially true of food waste, food packaging, used hygiene materials, as well as pet droppings. It has been shown that the number of microorganisms in the MSW can reach 10^8^–10^9^ CFU g^−1^. Therefore, they are a potential source of air, leachate and soil pollution in landfills.

It should be noted that there are currently no legal regulations in the world that would specify the limits of individual groups of microorganisms in the atmospheric air, leachate and soil in a landfill. Therefore, assessing this type of contamination is a difficult task and should be performed, taking into account the specifics of each place. There is little data in the literature on microbial contamination in illegal landfills. Thus far, such analyses have been performed for many MSW management facilities, such as sewage treatment plants [23,24], composting plants and other biomass processing facilities [25,26], waste sorting plants [27,28] and legal landfills [29,30].

Nair published a summary of microbial air pollution in landfills worldwide (Asia, Africa, Europe, North America, South America) [29]. In Poland, from municipal landfills areas (Sosnowiec and Janiki), the concentration of bacteria ranged from 7.0 × 10^1^ to 4.0 × 10^4^ CFU m^−3^ and fungal aerosol was from 2.0 × 10^2^ to 1.2 × 10^4^ CFUm^−3^ [31]. On the other hand, Breza-Boruta tested aerosol on the Municipal Waste Disposal Complex premises at Zolwin-Wypaleniska and Municipal Waste Treatment Plant in Inowrocław in Poland [4,32]. The author showed a large variation in the number of microorganisms in the air depending on the place of the study and the month in which the samples were taken. The number of bacteria in these studies was: 7.8 × 10^1^–5.4 × 10^5^ CFU m^−3^, *Pseudomonas fluorescens:* 0 to 4.3 × 10^2^ CFU m^−3^; mannitol-positive staphylococci: 0 to 4.9 × 10^3^ CFU m^−3^; Actinomycetes: 4 × 10^0^ to 2.3 × 10^4^ CFU m^−3^, *Enterobacteriaceae* 0 to 2.8 × 10^2^ CFU m^−3^ and fungi 0 to 2.2 × 10^4^ CFU m^−3^ [4,32]. The results obtained in the present study are in line with the previously published ranges of the number of microorganisms isolated from legal landfills. The fact that the number of bacteria in the air around the MSW was statistically significantly higher than in the control air indicates that the tested landfill is the source of the emission of these microorganisms to the atmosphere.

There is also little data in the literature on the number of microorganisms in leachates and soils at illegal MSW disposal sites. Most of the work is focused on chemical pollutants, including heavy metals [6].

Lemanowicz et al. showed that in the soil (0–20 cm) at such places, there is 3.6 × 10^6^–8.6 × 10^6^ CFU g^−1^ of bacteria, 6.7 × 10^4^–2.4× 10^6^ CFU g^−1^ of Actinomycetes and 8.9 × 10^4^–1.3 × 10^5^ CFU g^−1^ of fungi [14]. In turn, Hrenovic et al. studied total heterotrophic bacteria from 8 illegal dump-sites in Croatia [33]. The results obtained in the present study show a lower number of fungi than previously described in the literature [14]. This may be related to the specifics of the areas from which the samples were collected—Lemanowicz et al. studied landfills located in the forest, where the soil contains a lot of plant organic matter that favours the multiplication of fungi [14].

Mor et al. emphasised a high number of total coliform and faecal coliform in leachate and groundwater at a landfill site in India [34]. Likewise, Aderemi et al. analysed different locations adjacent to a MSW landfill [35]. They found that the total count of the *Enterobacteriaceae* in the leachate was 1.3 × 10^5^ CFU mL^−1^ while the total viable count of *Enterobacteriaceae* in the groundwater samples ranged from 4.0 × 10^3^–1.0 × 10^6^ CFU mL^−1^. Further, in the present study, bacteria from the *Enterobacteriaceae* family were detected in the leachate but in a lower number at the level of 10^1^–10^3^ CFU mL^−1^. The presence of lower faecal contamination in illegal landfills compared to municipal facilities seems logical.

### 3.3. Biodiversity of Air, Leachate and Soil

**Figure 7:**
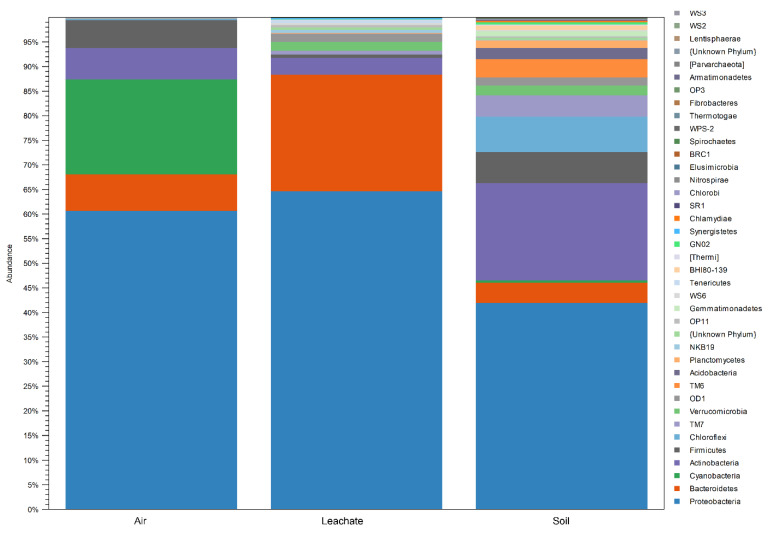
Percentage of identified bacteria at the Phylum level based on the analysis of the V3/V4 region of the gene encoding the 16S rRNA.

The percentage of identified bacteria at the Phylum level based on the analysis of the V3/V4 region of the gene encoding 16S rRNA is summarised in Figure 7, and detailed data on the phylogenetic affiliation of bacteria in the tested samples are included in Table S4 (Supplementary Materials).

Bacteria belonging to Proteobacteria dominated in all tested samples, constituting from 42% (soil) to 64.7% (leachate) OTU (Operational Taxonomic Unit). Relatively high relative abundance was also found for Bacteroidetes, from 4.2% (soil) to 23.7% (leachate); Actinobacteria, from 3.4% (leachate) to 19.8% (soil); and Firmicutes, from 0.7% (leachate) to 6.3% (soil). In addition, bacteria belonging to Cyanobacteria (19.3%) dominated in the air, OD1 (1.6%) and Verrucomicrobia (1.8%) in leachate, and Chloroflexi (7.2%), Gemmatimonadetes (1.0%), Planctomycetes (1.6%), TM6 (3.7%), TM7 (4.3%) and Verrucomicrobia (2.0%) in the soil (Figure 7).

When analysing bacteria at the class level, the dominance of Gammaproteobacteria (for air, leachate and soil: 50.7%, 26.2% and 23.3%, respectively), Alphaproteobacteria (6.5%, 12.8% and 8.3%), Betaproteobacteria (3.6%, 25% and 8.5%), Actinobacteria (6.3%, 3.1% and 14%) and Flavobacteriia (6.9%, 14.7% and 26.0%).

Moreover, bacteria of the Chloroplast (19.0%) and Bacilli (5.6%) classes were present with high relative abundance in the air. Rhodothermi (2.8%), Saprospirae (1.9%), Sphingobacteriia (4.2%) and Verrucomicrobiae (1.3%) had a high relative abundance in the leachate.

On the other hand, the soil showed an additional dominance of bacteria from the classes Acidobacteria (1.3%), Anaerolineae (1.9%), TK17 (1.15%), Clostridia (3.7%), Planctomycetia (1.3%), Deltaproteobacteria (1.8%) and SJA-4 (3.7%), TM7-1 (1.8%) and TM7-3 (2.2%) (Table S4).

The presence of bacteria belonging to a total of 78 orders was recorded in the air in the landfill area; greater diversity of bacteria was found in the leachate—121 orders, and the highest in the soil—204 orders (Table S4).

The analysis of the biodiversity of bacteria based on the sequence analysis of the V3/V4 hypervariable region of the gene encoding the 16S rRNA showed that the samples collected at the landfill were dominated by the following genera of bacteria: *Pseudomonas* (from 1.3 in the leachate to 4.4% in the air) and an unknown genera from family *Sinobacteraceae* (from 2.6% in the air to 4.9% in the soil). In addition, the following genera occurred in the air with a large share: *Escherichia* (39.6%), *Flavobacterium* (6.3%), *Bacillus* (4.4%), *Grevillea* (3.6%), *Stenotrophomonas* (2.5%), unknown genera from of the order Streptophyta (18.4%), Burkholderiales (2%) and unknown genera from the family *Micrococcaceae* (2.9%).

The following bacteria had a significant share in the leachate: *Aequorivita* (4.8%), *Perlucidibaca* (3.13), *Balneola* (2.8%), *Tenacibaculum* (2.8%), *Alcanivorax* (2.5%), *Fluviicola* (1.6%), HTCC (1.5%), *Verrucomicrobium* (1.4%), *Parvibaculum* (1.1%), unknown genera from *Idiomarinaceae* (7.4%), *Flavobacteriaceae* (4.0%), *Rhodobacteraceae* (2.2%), *Alcaligenaceae* (1.3%), OM60 (1.2%), *Sphingobacteriaceae* (1.7%), unknown genera from MKC10 order (9.9%), Saprospirales (1.4%), Sphingobacteriales (1.4%), Rhizobiales (1.3%) and an unknown genus from the class of Alphaproteobacteria (1.9%).

In turn, bacteria of the genera dominated the soil: *Cellulosimicrobium* (7.3%), *Escherichia* (3.6%), *Clostridium* (2%), *Perlucidibaca* (1.7%), *Bacillus* (1.2%), *Cellvibrio* (1, 1%), *Candidatus* Xiphinematobacter (1.1%), unknown genera from the family *Kineosporiaceae* (2.7%), *Xanthomonadaceae* (2.1%), *Rhodospirillaceae* (1.6%) and *Comamonadaceae* (1.2%).

Unknown genera from the orders Acidimicrobiales (4.5%), GCA004 (1.9%), IS-44 (1.2%), MND1 (2.4%) and classes of Gitt-GS-136 (3.6%), SJA-4 (3.6%), TM7-3 (2.2%), Betaproteobacteria (1.8%), TM7-1 (1.8%) and TK17 (1.1%). The remaining identified species in the tested samples accounted for <1%.

In the present study, we used high-throughput sequencing on Illumina MiSeq for assessing the diversity of microorganisms in the air, soil and leachate samples from illegal dumping sites.

The analysis of these samples allowed for a comprehensive study of the diversity of microorganisms in the dumpsite environment. Until now, only metagenomic analysis of soil and leachate from MSW management facilities has been published. It is worth emphasising that the conducted metagenomic analysis with high-throughput sequencing on Illumina MiSeq in the present study was performed for the first time for the air from illegal landfills.

This analysis showed a dominance of Proteobacteria, Actinobacteria, Bacteroidetes and Firmicutes in all types of tested samples. Additionally, in the air with high abundance was Cyanobacteria, and in the soil was Chloroflexi, TM7, TM6, and Verrucomicrobia.

Firmicutes, Proteobacteria, Bacteroidetes and Actinobacteria were the dominant phyla in landfills identified in previous studies in samples of cover soil, stored MSW, leachate and sediments [36,37,38].

Bacteria belonging to the Firmicutes phylum have cellulose-degrading abilities. These bacteria can play an important role in the anaerobic and methanogenic waste decomposition process. Simultaneously, Firmicutes have a leading share in the synthesis of fulvic-like substances and their humification [37,39]. Similarly, the main function of Bacteroidetes is a decomposition of starch polysaccharides and hydrolytic cellulose, while Proteobacteria are responsible for decomposing soluble sugars into monosaccharides and short-chain fatty acids [37,39]. Liu et al. indicate that Proteobacteria can promote aromatic compounds and alkoxy chain hydrocarbons with long branch chains and fever branches at both intermediate and old landfill stages [40].

Actinobacteria can play an essential role in the rapidly degrading simple lipids, proteins and carbohydrates [37].

Moreover, Cyanobacteria, Chloroflexi, Verrucomicrobia and TM6 belong to phyla identified earlier from landfills [39,41].

Phylum TM7 (known as Saccharibacteria) is ubiquitous and has been detected in soils, sediments and wastewater sludge. It also can be associated with human diseases, including periodontitis and inflammatory bowel disease [42]. There were suspicions that TM7 was involved in the degradation of toluene and benzene, but Figueroa-Gonzalez et al. ruled it out [43].

Microbial taxonomic analysis at the genus level showed the highest abundance of genera *Pseudomonas* in all types of tested samples (air, soil, leachate). *Pseudomonas* sp. is widely detected in various environments, including landfills [31,39]. These bacteria utilise a wide range of polycyclic aromatic hydrocarbons and have roles in degrading organic matter and denitrification [44,45,46].

*Bacillus* sp. and *Escherichia* sp. were detected in high abundance in the air and soil. They are common bacteria present in landfill environments. *Bacillus* is known for cellulose degradation, polycyclic aromatic hydrocarbons and chromium oxidation [47,48]. Threedeach et al. pointed out that up to 87.5% of the *Escherichia coli* isolated from landfill environments can be resistant to antibiotics, especially doxycycline, cephalothin, tetracycline and minocycline [49].

Among the identified bacteria were Aequorivita, Balneola, Cellvibrio, Clostridium Flavobacterium, Fluviicola, Stenotrophomonas, Parvibaculum, Perlucidibaca and Verrucomicrobium, previously described as characteristic of sites associated with MSW disposal [39,41,50,51].

However, also found with a large abundance were *Alcanivorax*, *Candidatus Xiphinematobacter*, *Cellulosimicrobium* and *Tenacibaculum*, which had not been previously reported for the test environment.

*Alcanivorax* is an isolated aquatic bacterium that plays an important role in the natural bioremediation of oil spills worldwide [52,53]. Likewise, bacteria of the genus *Tenacibaculum* constitute one of the dominant heterotrophic bacterial groups in aquatic habitats. *Tenacibaculum* can cause bacterial fish diseases such as enacibaculosis or flexibacteriosis [54].

*Candidatus* Xiphinematobacter is an endosymbiont from the dagger nematode *Xiphinema americanum*, a migratory ectoparasite of numerous crops [55].

*Cellulosimicrobium* is widely distributed in the environment and has been isolated mainly from the soil, water and grass cuttings. These bacteria have bioremediation capability for iron, zinc, copper, nickel and cadmium. Further, they have been reported for utilising benzo(a)pyrene, the sole carbon and energy source reducing toxic Cr(VI) to non-toxic Cr(III) and the remediation of thorium (IV) [56].

*Cellvibrio* has been reported as a degrader of cellulose, hemicellulose, dextran, xylan, chitin, starch and plant cell walls [57].

Notably, the literature describes the ability of the bacteria *Pseudomonas* and *Tenacibaculum*, which dominated in the landfill, to degrade polyester [58]. Moreover, *Alcanivorax* can form thick biofilms on low-density polyethene (LDPE) specifically and degrade petroleum-based plastic [59]. Additionally, *Parvibaculum* can degrade alkanes and alkynes. The domination of these bacteria in the landfill indicates that the microbiota has adapted to the environment where plastic MSW was deposited.

It is worth emphasising that of the identified bacteria, there may be potential human pathogens of the genera *Bacillus*, *Clostridium*, *Cellulosimicrobium*, *Escherichia* and *Pseudomonas* [60,61].

**Figure 8:**
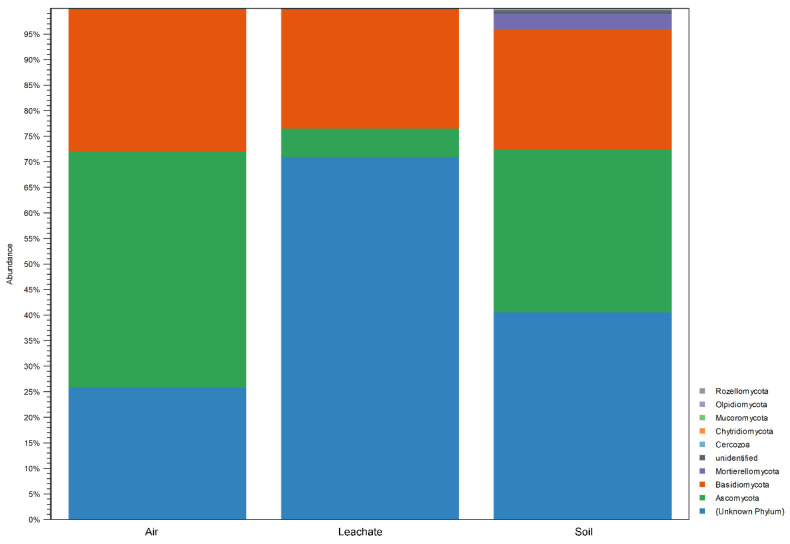
Percentage share of identified fungi at the Phylum level based on the ITS region analysis.

The percentage share of identified fungi at the Pylum level based on the analysis of the ITS region is summarised in Figure 8. Table S5 (Supplementary Materials) presents the correctly identified bacteria at the species level, along with their complete taxonomy and the percentage share in air, leachate and soil samples collected from the dumpsite.

In all the samples tested, fungi belonging to Basidiomycota dominated, constituting 23.3% (leachate) to 27.7% (air). Relatively high relative abundance was also found for Ascomycota—from 5.6% (leachate) to 46.3% (air). Moreover, fungi belonging to Mortierellomycota had a high share (3.1%) in the soil. It is noteworthy that for 15.9% of fungi from the air, 40.6% from the soil and 71.0% from the leachate, it was impossible to determine the taxonomic affiliation at the Phylum level (Figure 8). When analysing fungi at the class level, the dominance was by Eurotiomycetes (for air, leachate and soil: 2.2%, 1.8% and 4.2%, respectively), Sordariomycetes (1.5%, 1.2% and 16.3%), Tremellomycetes (13.6%, 15.3% and 14.7%). Moreover, in the air with high relative abundance, there were fungi of the classes Dothideomycetes (22.6%), Leotiomycetes (16.4%), Malasseziomycetes (8.6%), Agaricomycetes (4.7%) and Pezizomycetes (2.5%). Saccharomycetes (1.2%) and Malasseziomycetes (6.8%) had a high OTU in the leachate. On the other hand, additional dominance of fungi from the Agaricomycetes (7.8%), Mortierellomycetes (3.1%) and unidentified Ascomycota (9.6%) classes were detected in the soil (Table S5).

In the air in the landfill, the presence of fungi belonging to 62 orders was noted, the lower diversity of fungi was demonstrated for the soil of 54 orders, and the lowest for the leachate—22 orders (Table S5). The analysis of the biodiversity of fungi based on the sequence analysis of the ITS region showed that the samples collected at the landfill were dominated by species belonging to the genera: *Malassezia* (from 1.0% in the soil to 8.5% in the air) and *Bullera* (from 12.9% in the air to 15.0% in leachate). In addition, there were types in the air with a high relative abundance: *Blumeria* (12.3%), *Didymella* (6.9%), *Torula* (3.9%), *Alternaria* (3.8%), *Mycosphaerella* (3.4%), *Botrytis* (2.5%), *Peziza* (1.8%), *Aspergillus* (1.6%), *Cladosporium* (1.4%), *Strobilurus* (1.2%) and *Lycoperdon* (1.1%).

Fungi of the genus *Candida* (1.2%) had a significant share in the leachate. In turn, the soil was dominated by fungi of the genera: *Coprinellus* (6.2%), *Fusarium* (5.4%), *Microascus* (4.6%), *Mortierella* (3.1%), *Stachybotrys* (2.0%), *Chrysosporium* (1.6%) and *Aspergillus* (1.4%). Unknown species from the order Agaricales (1.2%) and an unknown species from Phylum Ascomycota (9.6%) were also detected with a high percentage share. Other identified species in the tested samples accounted for <1%.

Little is known about the diversity of fungi in landfills. The present work has detected the dominance of Basidiomycota, Ascomycota and Mortierellomycota.

Some of the identified species have previously been described as typical for environmental landfills: *Aspergillus*, *Alternaria*, *Candida*, *Cladosporium*, *Chrysosporium*, *Didymella*, *Fusarium* and *Stachybotrys* [29].

In addition, research conducted around the world has shown the presence of fungi of *Acremonium*, *Aureobasidium*, *Arthrographis*, *Curvularia*, *Drechslera*, *Epicoccum*, *Geotrichum*, *Mortierella*, *Myrothecium*, *Penicillium*, *Phoma*, *Tritirachium*, *Rhizopus*, *Sclerotinia*, *Staphylotrichum* and *Torula*, which have not been found in the current study [29,32,40,62]. However, the genera *Bullera*, *Blumeria*, *Coprinellus*, *Lycoperdon*, *Malassezia*, *Microascus*, *Mycosphaerella*, *Peziza* and *Strobilurus* were not previously indicated as typical for landfills.

*Blumeria* is phytopathogens of cereals and grasses [63,64].

Similarly, *Mycosphaerella* includes plant pathogens, saprobes and mutualistic fungi [65]. *Bullera* is an anamorphic form of *Tremellaceae*, which are cosmopolitan parasites of other fungi [66].

*Coprinellus*, *Lycoperdon*, *Peziza* and *Strobilurus* are saprobic cup fungi found throughout mainland Europe. *Coprinellus* is a wood-rotting fungus that grows on tree stumps and dead roots [67]. *Lycoperdon* occurs in all kinds of woodland, where they grow on the ground in leaf litter; also, less commonly, in permanent pasture. *Peziza* and *Strobilurus* occur mainly in the forest, often among gravel or shale [66]. The presence of these fungi is most likely related to the surroundings of the studied landfill and not the MSW.

*Microascus* (*Scopulariopsis* teleomorphs) are commonly isolated from soil, air, plant debris, paper, dung and moist indoor environments. These fungi can be opportunistic pathogens, primarily causing superficial tissue infections. Further, they are one of the principal causes of nondermatophytic onychomycoses [68].

Moreover, some other identified fungi are potentially hazardous to human health.

*Chrysosporium* are common soil saprophytes; however, they may cause skin infections, and onychomycosis in humans has occasionally been isolated from systemic infections [69]. Likewise, few *Candida* species are human opportunistic pathogens and cause superficial or invasive infections in the case of weakened or reduced human immunity [70].

*Malassezia* fungi are associated with various conditions, including dandruff, atopic eczema dermatitis, pityriasis versicolor, seborrheic dermatitis and folliculitis. *Malassezia* can also cause systemic infections in immunocompromised patients [71].

Moreover, *Aspergillus*, *Alternaria*, *Fusarium* and *Stachybotrys* are allergenic and toxinogenic moulds. Additionally, the allergenic effect is also assigned to *Cladosporium* and *Didymella* [72,73].

### 3.4. Cytotoxicity of Soil Extracts and Waste Leachate Samples Assessment

The justification for selecting such cell lines for current research was as follows. The A-549 lung cell line was selected for its frequent use in assessing the cytotoxicity of dust and air pollutants [74]. The intestinal Caco-2 cell line because part of the inhaled dust can be swallowed with air, reach the gastrointestinal tract (GIT) and affect the intestinal epithelium, resulting in colitis [75]. The normal intestinal IEC-6 line was selected for the same reason. The Hep-G2 liver cell line was also applied, as different toxicants, odours and ingested dust can cause oxidative stress in the liver and lead to inflammation and DNA damage [76].

**Figure 9:**
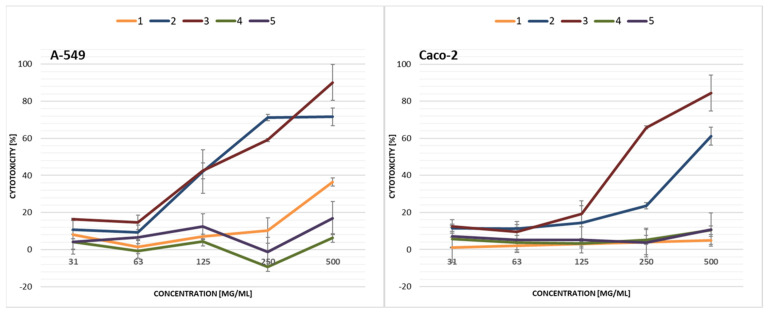
Cytotoxicity of municipal solid waste leachate samples collected from the landfill in the *PrestoBlue* assay after 72 h exposure of A-549 lung and Caco-2 intestinal epithelial cells. Each data point represents the mean of the fluorescence values from the cells of the four replicates. Results are presented as mean ± SD.

First, the cytotoxicity of the collected samples was screened against A-549 and Caco-2 cells (Figure 9). Leachate samples 2 and 3 demonstrated the strongest cytotoxicity against both A-549 and Caco-2 cells, while A-549 cells showed greater sensitivity towards the tested samples than Caco-2 cells. For sample 2, the IC_50_ values were 159 (A-549) and 426 (Caco-2) mg/mL, while for sample 3 it was 182 (A-549) and 208 (Caco-2) mg/mL, respectively (Table 3).

**Figure 10:**
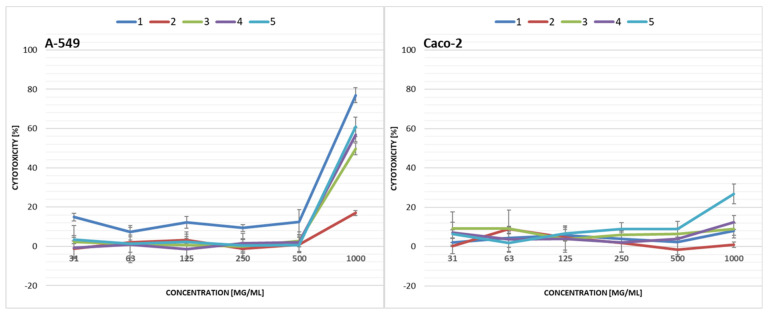
Cytotoxicity of soil extracts collected from the landfill in the *PrestoBlue* assay after 72 h exposure of A-549 lung and Caco-2 intestinal epithelial cells. Each data point represents the mean of the fluorescence values from the cells of the four replicates. Results are presented as mean ± SD.

Both cell lines (i.e., A-549 and Caco-2) were less sensitive to the soil extracts than to the leachate samples. Caco-2 cells displayed the highest resistance to soil extracts to the extent that no IC_50_ concentration could be determined for any (Figure 10). For A-549 cells, IC_50_ values were determined for samples 6, 9 and 10 (Table 3). However, only the stock extracts (1000 mg/mL) showed cytotoxicity up to a maximum approx. 78% ± 3.83%.

After screening the cytotoxicity on A-549 and Caco-2 cells, the most cytotoxic samples were selected for further testing on Hep-G2 and then also for IEC-6 cells. These were MSW leachate samples 2, 3 and 5, and soil extract 5, respectively. Dose-dependent curves for these findings are not shown; only the IC_50_ values in Table 3 are presented. IEC-6 cells were more sensitive to the MSW leachate samples than Hep-G2 cells, while the opposite was for soil extract 5 in the case of IEC-6 cells, IC_50_ value could not be determined (Table 3). After analysing the results presented in Table 3, it can be concluded that leachate samples 2 and 3 were the most cytotoxic to cancerous Caco-2 cells, then Hep-G2, then A-549, while normal IEC-6 intestinal cells exhibited up to nine times greater sensitivity.

### 3.5. Genotoxicity of Seepage Leachate and Soil Samples

As a result of the cytotoxicity screening of leachate samples and soil extracts from the landfill, only those samples for which IC_50_ values could be determined were selected for genotoxicity testing. Because the tested samples were weakly cytotoxic to Caco-2 cells, the genotoxicity analysis was performed only on A-549 cells. Concentrations close to and below the IC_50_ values were selected. The results are summarised in Table 4 and Table 5. The genotoxicity of the negative control (cells not exposed) was 5.4 ± 0.5%, while that of the positive control (cells exposed to 25 µM H_2_O_2_) was 27.8 ± 3.6%. In general, the tested leachate samples were characterised as no genotoxic (concentration 125 mg/mL) or medium or weakly genotoxic (concentration 250 mg/mL) (Table 4). Soil extracts showed even lower genotoxicity. The highest genotoxicity was obtained for sample 4, with a concentration of 1000 mg/mL (14.5 ± 3.5%) (Table 5).

### 3.6. Targeted and Untargeted UPLC-HRMS Analysis

The results obtained in the process of the identification of toxic compounds in leachate and soil from illegal landfills are summarised in Table 6.

The targeted identification of pesticides and other contaminants commonly found in environmental water was performed using the TargetScreener (Bruker Daltonics) method that is based on tight tolerances matching analyte retention time, precursor *m*/*z*, fragments *m*/*z*, precursor and fragments isotopic pattern and precursor-to-fragment signals intensity ratios. Detailed data on identifications are provided in Table S6 (Supplementary Materials).

As can be seen in Table 6, both soil and leachate samples contained common pesticides: carbamazepine, prometryn, terbutryn, permethrin, carbanilide, pyrethrin, carbaryl and prallethrin. The list also contains quaternary ammonium compounds: benzalkonium chlorides: BAC14 and BAC18, which are commonly used surfactants and biocides. Among identifications, there were also large scale produced chemicals and/or polymer degradation products, such as melamine, triphenylphosphate, diphenylphtalate, insect repellent diethyltoluamide, and also the common drug ketoprofen. It was demonstrated that several of these chemicals could induce cytotoxicity in numerous cell lines: diclofenac in breast (MCF-7), cervical (HeLa) and colorectal (HT-29) cancers [77,78]; ketoprofen in the human embryonic kidney (HEK 293) [79]; DDAC-C10 in lung cancer (A-549) [80]; melamine in HEK 293, fibroblasts (L929) and Chinese hamster ovary (CHO) [81,82]; carbaryl in human umbilical vein endothelial cells (HUVEC) and rat adrenal pheochromocytoma (PC-12) [83,84]. In our studies, leachate no. 2 contained the most (i.e., 9) cyto and genotoxins, the chemical residues detected are presented in Table 6.

### 3.7. Statistical Analysis of LC-MS Data

**Figure 11:**
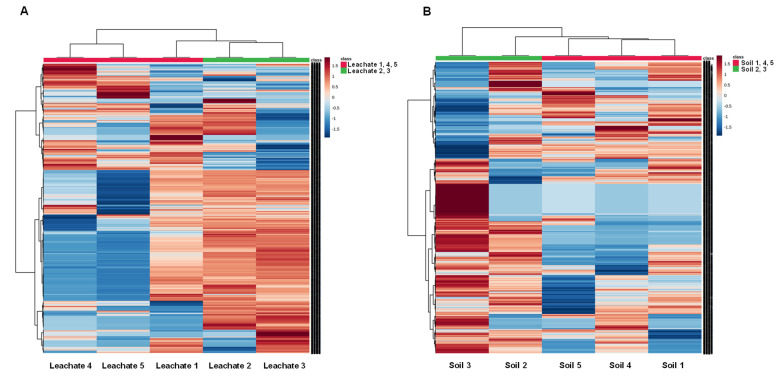
Hierarchical clustering heatmaps representing MS features are significantly (*p* < 0.05) different between groups for (**A**) leachate and (**B**) soil samples. The analysis was performed using an Euclidean distance measure and Ward’s distance measure algorithm; The rows display *m*/*z* values, and the columns represent samples. The blue colour of the tile indicates low abundance, and red indicates high abundance.

The comparative analysis of MS features further investigated the chemical composition of leachate samples and soil type. The much higher cytotoxicity of leachate and soil samples marked with numbers 2 and 3 compared to samples numbers 1, 4 and 5 was the reason behind the comparison of the mentioned two groups performed in MetaboScape 2021 and then in MetaboAnalyst 5.0. Heatmaps were created to visualise the differences in the MS features between the soil and leachate samples from different sampling sites (Figure 11). Heatmaps were created for the top 1000 *m*/*z* values selected based on their interquartile range (IQR) that differed significantly between the two groups of samples. Heatmaps clearly show that there is a high similarity between samples 2 and 3 (green) and 1, 4 and 5 (red) in both cases. In the case of leachates, samples 2 and 3 seem to be similar to sample number 1, which came from the same area of an illegal landfill.

Furthermore, much more MS features were identified in these groups than in samples 4 and 5. A similar trend can be observed in the soil samples. Comparisons of MS features in soil samples indicate that soil 5 appears associated first with both soil 1 and 4 and then with soil sample numbers 2 and 3.

**Figure 12:**
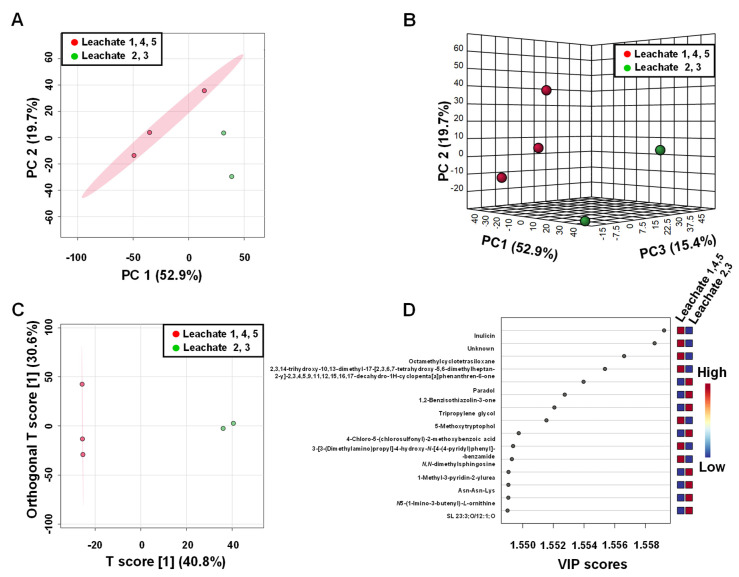
Analysis of leachate compound profiles created for LC-MS data. (**A**) 2D PCA; (**B**) 3D PCA; (**C**) OPLS-DA scores plots generated from the LC MS data of leachates 2, 4 and 5 (red) and leachates 2 and 3 (green); (**D**) Metabolite ranking for the OPLS-DA model according to VIP scores.

Univariate and multivariate statistical analyses of MS features were employed to examine the relationships between two groups of the soil and leachate samples from different areas of illegal landfills. MS features following normalisation by sum, log-transformation and auto-scaling were analysed with principal component analysis (PCA) to assess whether samples 2 and 3 versus samples 1, 4 and 5 could be separated based on distinct MS profiles. The resulting 2D and 3D PCA scores plots (Figure 12A,B) showed that the MS features profiles of leachate samples 2 and 3 are, to a large extent, dissimilar from leachate samples 1, 4, 5, with PC1 and PC2 accounting for 52.9% and 19.7% of the variance, respectively.

Group separations were also inspected using OPLS-DA, which revealed a clear separation between these two groups of leachate samples (Figure 12C). In order to evaluate the statistical robustness of this model, 2000 permutation tests were conducted. Decent discrimination was observed between the two groups of the samples (Q^2^ = 0.987, R^2^Y = 0.998, *p*-value < 0.05 (0/2000)), revealing substantial differences in MS features profiles of leachates 2 and 3 and samples 1, 4 and 5 (Figure S1, Supplementary Material).

An OPLS-DA statistical method referred to as S-plot was employed to identify MS features present in significantly different levels in both groups of samples. Higher values of |p(corr)| indicate more important values to the classification. Variables with a |p(corr)| value greater than 0.5 were considered significant. A total of 406 variables were negatively correlated to group separation, showing a p(corr) score of <−0.5 and 1360 variables were positively correlated to group separation showing a p(corr) score of >0.5. A paired *t*-test was performed for all variables. An analysis of the S-score of the OPLS-DA model, combined with independent statistical *t*-test analysis (*p* values < 0.05), indicated that these 1766 MS features were responsible for the differences. Based on the fold change analysis (FC), we narrowed down this group to 1364 MS features with a fold change with FC ≥ 2 or ≤ 0.5 (|log_2_ FC| ≥ 1). In order to confirm the correlativity of each selected MS feature, we performed variable influence on projection (VIP) for the OPLS model. All selected *m*/*z* values have a VIP value above 1.0. The first 15 MS features with the highest VIP values are shown in Figure 12D. Out of the 1364 *m*/*z* values, which differ to the greatest extent between the two groups of leachate samples, compound name annotations were made for 784 MS features using MS and MS/MS data (Table S7, Supplementary Materials).

**Figure 13:**
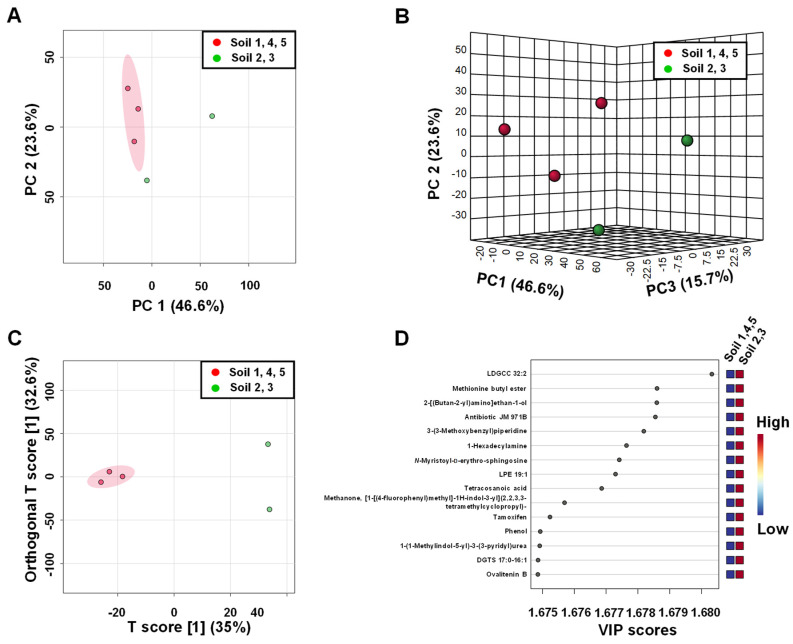
Analysis of soil profiles created for LC-MS data. (**A**) 2D PCA; (**B**) 3D PCA; (**C**) OPLS-DA scores plots generated from the LC MS data of the soil 2, 4 and 5 (red) and soil 2 and 3 (green) samples; (**D**) Metabolite ranking for OPLS-DA model according to VIP scores.

Chemical compounds abundances obtained from LC-MS experiments on soil samples were also subjected to statistical data analysis A 2D and 3D PCA scores plot to reveal good discrimination between soil samples with higher and lower cytotoxicity (Figure 13A,B). Comparing soil samples 2 and 3 versus 1, 4 and 5 with OPLS-DA revealed excellent discrimination between these two groups (Figure 12C). The quality factors for this model were: Q^2^: 0.982 and R^2^Y: 0.992, with *p*-values based on permutation tests lower than 0.05 (Figure S2, Supplementary Materials). The analysis of S-plots revealed that 1014 MS features were positively correlated with group separation with a p(corr) score >0.5, and 397 features values were negatively correlated with a p(corr) score < −0.5. Fold change analysis indicated 1525 MS features with a fold change with FC ≥ 2 or ≤ 0.5 (|log_2_ FC| ≥ 1). The VIP plot for the OPLS model indicated that all selected *m*/*z* values have a VIP value above 1.0. The first 15 MS features with the highest VIP values are shown in Figure 13D. Furthermore, all data sets were further subjected to independent *t*-test analysis, which indicated that all selected MS features were statistically significant with *p*-values below 0.05. Out of the 1525 features that differ to the greatest extent between the two groups of soil samples, annotations were made for 611 MS features using MS and MS/MS data.

### 3.8. Pathway Analysis

The most differentiating compounds identified in soil and leachate samples number 2 and 3 vs. 1, 4 and 5 were subjected to pathway analysis in MetaboAnalyst 5.0. In this study, we selected a library of twelve organisms derived from KEGG metabolic pathways, including all accessible microorganisms—yeast: *Saccharomyces cerevisiae*; bacteria: *Escherichia coli*, *Bacillus subtilis*, *Pseudomonas putida*, *Staphylococcus aureus*, *Thermotoga maritima*, *Mesorhizobium japonicum*, *Klebsiella pneumoniae*, *Klebsiella variicola* and *Streptococcus pyogenes*; cyanobacterium *Synechococcus elongatus*; and, additionally, parasite: *Schistosoma mansoni*; for pathway analysis.

**Figure 14:**
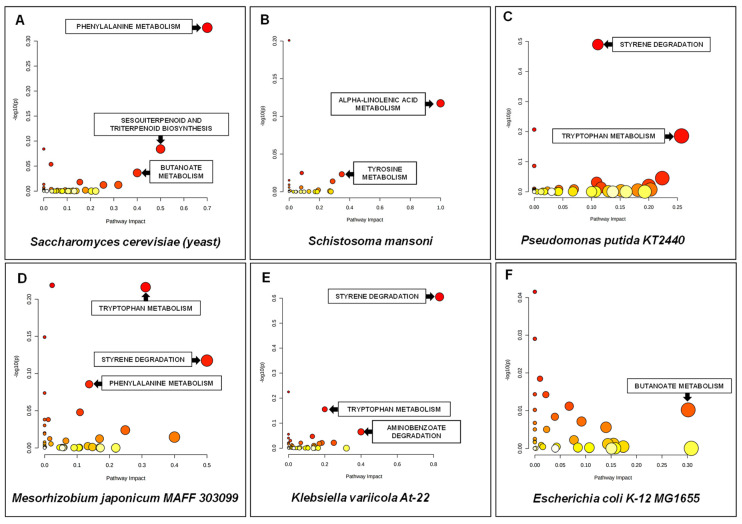
Summary of pathway analysis for leachate samples. All matched pathways are displayed as circles. The colour and size of each circle are based on the *p*-value and pathway impact value. Metabolites from: (**A**) *Saccharomyces cerevisiae*; (**B**) *Schistosoma mansoni*; (**C**) *Pseudomonas putida*; KT2440; (**D**) *Mesorhizobium japonicum* MAFF 303099; (**E**) *Klebsiella variicola* At-22; (**F**) *Escherichia coli* K-12 MG1655.

For the KEGG pathway enrichment analysis, 471 compounds were assigned to twenty-six metabolic pathways, including butanoate metabolism, α-linolenic acid metabolism, styrene degradation, tryptophan metabolism, phenylalanine metabolism, atrazine degradation, sesquiterpenoid and triterpenoid biosynthesis, glyoxylate and dicarboxylate metabolism, pyruvate metabolism and aminobenzoate degradation. The most significant results for the selected microorganisms are shown in Figure 14.

**Figure 15:**
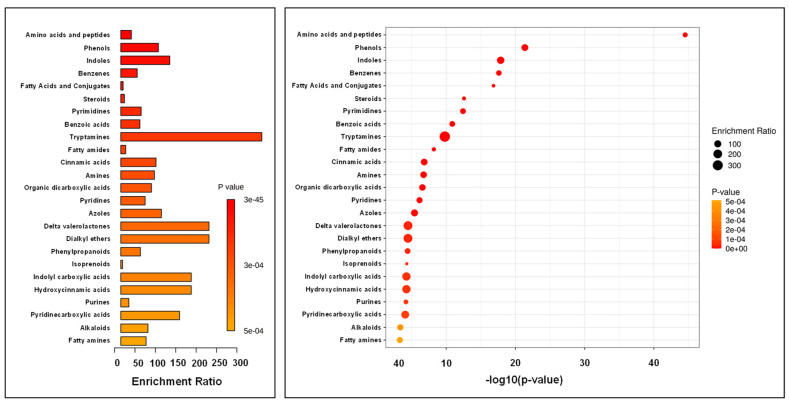
Summary of pathway enrichment analysis of leachate samples based on main class compound analysis. The most significant *p*-values are in red, while the least significant are in orange. The Enrichment Ratio is computed by Hits/Expected, where hits = observed hits; expected = expected hits.

In the second step, we performed pathway enrichment analysis of leachate samples based on the main class compounds analysis. The most significantly enriched main class turned out to be amino acids and peptides (Figure 15). Moreover, phenols, indoles and benzenes were also greatly differentiating classes.

In order to identify the pathways that are the most relevant in soil samples, all 614 annotated features were analysed. However, targeted pathway analysis was performed for 413 HMDB or KEGG ID compounds. Metabolic pathways with values >0.1 were considered to be significant. Twenty-six metabolic pathways, including one carbon pool by folate, phenylalanine metabolism, sphingolipid metabolism, atrazine degradation, sesquiterpenoid and triterpenoid biosynthesis, styrene degradation and aminobenzoate degradation with the highest impact values were significantly related to the differentiation between the above-discussed soil samples. The best results for the selected microorganisms are shown in Figure 14.

**Figure 16:**
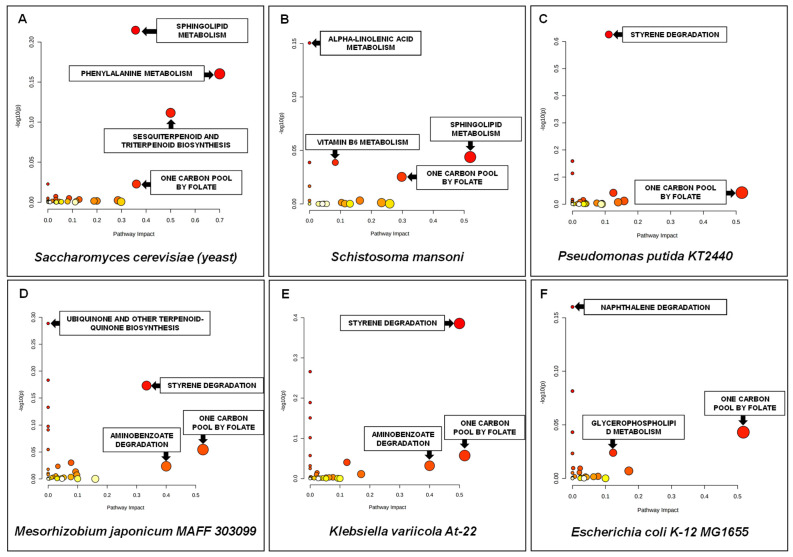
Summary of pathway analysis for soil samples. All the matched pathways are displayed as circles. The colour and size of each circle are based on the *p*-value and pathway impact value. Metabolites from: (**A**) *Saccharomyces cerevisiae*; (**B**) *Schistosoma mansoni*; (**C**) *Pseudomonas putida*; KT2440; (**D**) *Mesorhizobium japonicum* MAFF 303099; (**E**) *Klebsiella variicola* At-22; (**F**) *Escherichia coli* K-12 MG1655.

Pathway enrichment analysis of soil samples was also performed, focusing on main class compounds. The most significantly enriched main classes were benzene, amino acids and peptides (Figure 16). A significant number of steroids and benzoic acid were also considered as significantly differentiating the two soil sample groups.

**Figure 17:**
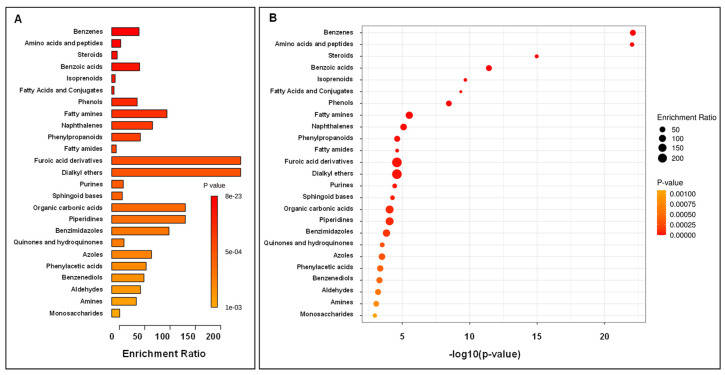
Summary of pathway enrichment analysis of soil samples based on main class compound analysis. The most significant *p*-values are in red, while the least significant are in orange. The Enrichment Ratio is computed by Hits/Expected, where hits = observed hits; expected = expected hits.

Pathway analysis showed that the compounds that differentiate the most soil and leachate samples with different cytotoxicity were associated with 13 common metabolic pathways, including aminobenzoate degradation, arginine and proline metabolism, atrazine degradation, biotin metabolism, glutathione metabolism, lysine degradation, phenylalanine metabolism, sesquiterpenoid and triterpenoid biosynthesis, sphingolipid metabolism, styrene degradation, sulphur metabolism, tryptophan metabolism and tyrosine metabolism (Figure 17). The results of metabolic pathway enrichment analysis showed that 21 main class compounds, common for soil and leachates, including fatty acids and conjugates, benzamides, steroids, amino acids and peptides, benzenes, sphingoid bases, benzoic acids, indoles, pyridines, phenylpropanoids, alcohols and polyols, amines, aromatic heteropolycyclic compounds, cinnamic acids, phenylacetic acids, alkaloids, piperazines, phosphate esters, organic carbonic acids, delta valerolactones and piperidinones were mainly involved in the toxicity of soil and leachate samples (Figure 17). Many of these compounds were classified by the International Agency for Research on Cancer as “carcinogenic to humans” (Group 1), “probably carcinogenic to humans” (Group 2A) or “possibly carcinogenic to humans” (Group 2B) [85].

It is worth recalling that isolates from extreme environments may show different metabolic activity than strains of the same species grown under standard conditions. Present databases are still too poor to connect directly detected metabolites with microorganisms in the studied environment. Metabolome data deposited in KEGH and HMDB included only ten bacteria and fungi metabolites, while almost 900 species were identified. The strains from the landfill where plastics were collected primarily will likely have a modified metabolism in terms of polymer degradation, which is confirmed by the results of the metabolomic analysis obtained in the present work.

## 4. Conclusions

The tested landfill is a source of physicochemical, biological and toxicological hazards for humans. Dominance (99.89% and 99.99%) shows particles with dimensions below 1 µm (PM_1_) in total airborne dust at the tested locations. The PM_1_ concentration is almost twice as high as the environmental threshold (0.025 mg m^−3^).

Quantitative analysis of the microbial contamination of air, leachate and soil fills the data gap on microbiological hazards caused by illegal landfills. Thus far, such analyses have been conducted most often for legally operating landfills or other facilities related to waste management (wastewater treatment plant, composting plant, waste sorting plant).

It was shown that the number of microorganisms in the air of the examined landfill is in line with the previously published ranges for legal landfills. The fact that the number of bacteria in the air around the MSW was statistically significantly higher than in the control air indicates that the tested landfill is the source of the emission of these microorganisms to the atmosphere.

Among identified microorganisms from tested landfills were bacteria (*Bacillus*, *Clostridium*, *Cellulosimicrobium*, *Escherichia*, *Pseudomonas*) and fungi (*Microascus*, *Chrysosporium*, *Candida*, *Malassezia*, *Aspergillus*, *Alternaria*, *Fusarium*, *Stachybotrys*, *Cladosporium*, *Didymella*) that are potentially hazardous to human health.

Cytotoxic activity was detected for 2 of 5 water samples and 3 of 5 soil samples from the landfill sites. Leachate samples 2 and 3 were the most cytotoxic to cancerous Caco-2 cells, then Hep-G2, then A-549, while normal IEC-6 intestinal cells exhibited up to nine times greater sensitivity. Soils 1, 4, and 5 were cytotoxic to A-549, while to Hep-G2, only soil sample 5 was cytotoxic. The toxic activity of the tested samples may be induced by the presence of pesticide and drug residues as well as their metabolites and/or metabolites of microorganisms detected in the tested samples. The cytotoxicity results of the leachate and soil samples are worrying because below the sampling site is a lake where people fish. Compounds responsible for cytotoxicity may also adversely affect fish, accumulate in them, and go further to other elements of the trophic chain (animals, humans). However, tested leachates and soils were characterised as no genotoxic or weakly genotoxic, except for the most cytotoxic leachate no. 2, the genotoxicity of which could be defined as “medium”.

Common pesticides (carbamazepine, prometryn, terbutryn, permethrin, carbanilide, pyrethrin, carbaryl and prallethrin), quaternary ammonium compounds (benzalkonium chlorides), chemicals and/or polymer degradation products (melamine, triphenylphosphate, diphenylphtalate, insect repellent diethyltoluamide), as well as a common drug (ketoprofen) were found in soil and leachate samples. Compounds from soil (471) and leachates (413) belonged to twenty-six metabolic pathways, including metabolisms of pesticides, herbicides and polymers. However, present databases are still too data poor for connecting directly detected metabolites with microorganisms present in the studied environment.

This paper shows that the dangers posed by illegal landfills should not be ignored. It is recommended to perform comprehensive analyses on this type of site, including detailed microbiological and toxicological analyses that would allow for a better understanding of the mechanisms and scale of the impact of illegal landfills on the environment.

Moreover, currently, there are no clear legal regulations regarding the assessment of risks caused by illegal landfills in Poland. In most cases, experts decide on the scope of analysis, considering the ratio of their costs to the expected risk. The presented study may constitute the basis for the development of guidelines for conducting this type of research, considering the need to perform microbiological (including air) and toxicological analyses, which are often omitted. Future work should seek to develop a critical workflow for assessing the risks posed by illegal landfills in cooperation with state administration authorities.

## Figures and Tables

**Table 1:** Characteristics of sampling sites in an illegal dumpling.

No.	Description	Sample No
A	The pile of MSW on the hill on the south side. Numerous streams of leachates. The leachates were black, and the water was flowing down the hill towards the ponds (clay pits) located approx. 12 m below. Water in ponds was class I purity for surface waters.	Air 1 Soil 1 Soil 2 Soil 3 Leachate 1 Leachate 2 Leachate 3
B	A roofed, open hall, remains of an old brick factory, with plastic MSW predominating.	Air 2
C	The pile of MSW on the hill on the west side is dominated by plastic MSW, below there are small ponds with visible places for catching fish. Green seepage water.	Air 3 Soil 4 Leachate 4
D	Plastic MSW dominates between the MSW piles on the northern side, yellow-green colour of the leachates.	Air 4 Soil 5 Leachate 5
E	The corner of the heap on the side of the access road, plastic MSW predominates, no leachates.	Air 5
F	250 m from tested landfill site (Internal control sample).	Air 6
G	10 km from tested landfill site (External control sample).	Air 7

MSW—municipal solid waste.

**Table 2:** Media used for microbiological contamination of air analysis.

Microorganism Type	Medium	Supplier
fungi	Malt Extract Agar (MEA) medium with (0.1%) chloramphenicol	Merck Life Science, Warsaw, Poland
xerophilic fungi	DG18 LAB-AGAR™ (DG18 Agar)	Biomaxima, Lublin, Poland
bacteria	Tryptic Soy Agar (TSA) with (0.2%) nystatin	Merck Life Science, Warsaw, Poland
haemolytic Staphylococcus	Columbia Blood Agar with (0.2%) nystatin	Oxoid, Dardilly Cedex, France
actinomycetes	Pochon’s agar with (0.2%) nystatin	Labomix, Łódź, Poland
mannitol-positive *Staphylococcus* spp.	Chapman Agar with (0.2%) nystatin	Merck Life Science, Warsaw, Poland
*Pseudomonas fluorescens*	King B medium with (0.2%) nystatin	Hi Media Laboratories, Mumbai, India
*Enterobacteriaceae*	Violet Red Bile Glucose Agar (VRBG LAB-AGAR) with (0.2%) nystatin	Biomaxima, Lublin, Poland

**Table 3:** IC_50_ (mg/mL) values of waste leachate samples and soil extracts collected from the landfill against cell lines.

Sample	Cell Line			
	A-549	Caco-2	Hep-G2	IEC-6
Leachate 1	nd *	nd	na **	na
Leachate 2	159	426	371	47
Leachate 3	182	208	222	33
Leachate 4	nd	nd	na	na
Leachate 5	nd	nd	na	na
Soil 1	791	nd	na	na
Soil 2	nd	nd	na	na
Soil 3	nd	nd	na	na
Soil 4	937	nd	na	na
Soil 5	909	nd	692	nd

* not detected; ** not analysed.

**Table 4:** Genotoxicity of the leachate sampled from the landfill to A-549 lung epithelial cells as % of DNA in the comet tail in the alkaline comet assay. Results presented as the mean of the 50 cells analysed for each concentration ± S.E.M. * Results significantly different from unexposed control (*p* < 0.05).

Sample	Genotoxicity (% DNA in Comet Tail) ± S.E.M.	
	Concentration (mg/mL)	
	250	125
Leachate 2	20.1 ± 3.5 *	3.5 ± 0.8
Leachate 3	14.0 ± 3.4 *	6.5 ± 1.1

**Table 5:** Genotoxicity of the soil extracts sampled from the landfill to A-549 lung epithelial cells as % of DNA in the comet tail in the alkaline comet assay. Results presented as the mean of the 50 cells analysed for each concentration ± S.E.M. * Results significantly different from unexposed control (*p* < 0.05).

Sample	Genotoxicity (% DNA in Comet Tail) ± S.E.M.	
	Concentration (mg/mL)	
	1000	500
Soil 1	10.0 ± 2.6	4.6 ± 1.5
Soil 4	14.5 ± 3.5 *	3.2 ± 0.5
Soil 5	9.4 ± 2.5	3.7 ± 1.2

**Table 6:** Toxic compounds found in leachate and soil samples with the TargetScreener measurement method.

Compound/ Sample	Leachate					Soil				
	1	2	3	4	5	1	2	3	4	5
Carbamazepine	+	+	+			+	+			
Diclofenac		+					+			
Prometryn							+			
Terbutryn										
Triphenylphosphate						+		+	+	+
Ketoprofen	+	+	+							
Permethrin				+						
Triphenylphosphate	+									
BAC 18				+						
BAC 14						+				
Carbanilide	+	+	+	+	+					
DDAC-C10	+	+		+	+	+	+	+	+	+
Diethyltoluamide	+	+		+	+				+	
Dimethylphthalate	+	+	+	+	+					
Melamine	+	+	+				+		+	+
Pyrethrin						+	+	+	+	
Carbanilide						+	+	+	+	+
Carbaryl		+								
Prallethrin				+						

## Data Availability

The data presented in this study are available on request from the corresponding author.

## Supplementary Materials

The following supporting information can be downloaded at: https://www.mdpi.com/article/10.3390/ijerph19084826/s1, Table S1: Microbiological contamination of the air in the tested landfill; Table S2: Microbiological contamination of the leachate in the tested landfill; Table S3: Microbiological contamination of the soil in the tested landfill; Table S4: Diversity of bacteria in landfill environment; Table S5: Diversity of fungi in landfill environment; Table S6: Identification parameters of toxic compounds found in leachate and soil samples; Table S7: Analysis of metabolites detected in leachate samples; Figure S1: OPLS-DA model validation of LC-MS data. (A) The permutation test showing the observed and cross-validated R2Y and Q2 coefficients based on 2000 permutations of metabolites from two groups of leachate samples with a statistically significant *p*-value < 0.05 (0/2000). (B) Model overview showing the R2X, R2Y and Q2 coefficients for the groups; Figure S2: OPLS-DA model validation of LC-MS data. (A) The permutation test showing the observed and cross-validated R2Y and Q2 coefficients based on 2000 permutations of metabolites from two groups of soil samples with a statistically significant *p*-value < 0.05 (0/2000). (B) Model overview showing the R2X, R2Y and Q2 coefficients for the groups.

## References

[1] (2021). Statistics Poland Environment. https://stat.gov.pl/en/topics/environment-energy/environment/environment-2021,1,13.html.

[2] (2018). Directive (EU) 2018/851 of the European Parliament and of the Council of 30 May 2018 amending Directive 2008/98/EC on waste. Off. J. Eur. Union.

[3] Number of Illegal Dumps Reported in Europe as of 2021 by Country. https://www.statista.com/statistics/990529/estimated-number-of-illegal-dumps-in-europe/#statisticContainer.

[4] Breza-Boruta B., Lemanowicz J., Bartkowiak A. (2016). Variation in biological and physicochemical parameters of the soil affected by uncontrolled landfill sites. Environ. Earth Sci..

[5] Malinowski M., Wolny-koładka K., Jastrzębski B. (2015). Characteristics of illegal dumping sites—Case study: Watercourses. Infrastrukt. I Ekol. Teren. Wiej..

[6] Du L., Xu H., Zuo J. (2021). Status quo of illegal dumping research: Way forward. J. Environ. Manag..

[7] Seror N., Portnov B.A. (2020). Estimating the effectiveness of different environmental law enforcement policies on illegal C&D waste dumping in Israel. Waste Manag..

[8] Yang W., Fan B., Desouza K.C. (2019). Spatial-temporal effect of household solid waste on illegal dumping. J. Clean. Prod..

[9] Cahill E. (2020). Sampling, Analysis, and Risk Assessment for Asbestos and Other Mineral Fibers in Soil. Environ. Eng. Geosci..

[10] Limoli A., Garzia E., De Pretto A., De Muri C. (2019). Illegal landfill in Italy (EU)—A multidisciplinary approach. Environ. Forensics.

[11] Kiessling T., Knickmeier K., Kruse K., Brennecke D., Nauendorf A., Thiel M. (2019). Plastic Pirates sample litter at rivers in Germany—Riverside litter and litter sources estimated by schoolchildren. Environ. Pollut..

[12] Golimowski J., Lisiewicz M., Gawrys M. (2003). Deposition of lead, cadmium, copper and zinc with sediment dust in the vicinity of a landfill. Ann. Warsaw Agric. Univ. L. Reclam..

[13] Akortia E., Olukunle O.I., Daso A.P., Okonkwo J.O. (2017). Soil concentrations of polybrominated diphenyl ethers and trace metals from an electronic waste dump site in the Greater Accra Region, Ghana: Implications for human exposure. Ecotoxicol. Environ. Saf..

[14] Lemanowicz J., Bartkowiak A., Breza-Boruta B. (2016). Changes in phosphorus content, phosphatase activity and some physicochemical and microbiological parameters of soil within the range of impact of illegal dumping sites in Bydgoszcz (Poland). Environ. Earth Sci..

[15] (2019). Workplace Exposure—Measurement of Airborne Microorganisms and Microbial Compounds—General Requirements.

[16] Ferris M.J., Muyzer G., Ward D.M., Spring O. (1996). Denaturing Gradient Gel Electrophoresis Profiles of 16S rRNA-Defined Populations Inhabiting a Hot Spring Microbial Mat Community. Appl. Environ. Microbiol..

[17] White T.J., Bruns S., Lee S., Taylor J. (1990). Amplification and direct sequencing of fungal ribosomal RNA genes for phylogenetics. PCR Protoc. Guid. Methods Appl..

[18] Jensen E.A., Berryman D.E., Murphy E.R., Carroll R.K., Busken J., List E.O., Broach W.H. (2019). Heterogeneity spacers in 16S rDNA primers improve analysis of mouse gut microbiomes via greater nucleotide diversity. Biotechniques.

[19] MassBank of North America (MoNA) https://mona.fiehnlab.ucdavis.edu/.

[20] National Institute of Standards and Technology Mass Spectrometry Data Center https://chemdata.nist.gov/.

[21] Pang Z., Chong J., Zhou G., de Lima Morais D.A., Chang L., Barrette M., Gauthier C., Jacques P.-É., Li S., Xia J. (2021). MetaboAnalyst 5.0: Narrowing the gap between raw spectra and functional insights. Nucleic Acids Res..

[22] European Council (2008). Directive 2008/50/EC of the European Parliament and of the Council of 21 May 2008 on ambient air quality and cleaner air for Europe. Off. J. Eur. Union.

[23] Szulc J., Okrasa M., Majchrzycka K., Sulyok M., Nowak A., Ruman T., Nizioł J., Szponar B., Gutarowska B. (2021). Microbiological and Toxicological Hazards in Sewage Treatment Plant Bioaerosol and Dust. Toxins.

[24] Xu P., Zhang C., Mou X., Wang X.C. (2020). Bioaerosol in a typical municipal wastewater treatment plant: Concentration, size distribution, and health risk assessment. Water Sci. Technol..

[25] Szulc J., Otlewska A., Okrasa M., Majchrzycka K., Sulyok M., Gutarowska B. (2017). Microbiological contamination at workplaces in a combined heat and power (CHP) station processing plant biomass. Int. J. Environ. Res. Public Health.

[26] Gutarowska B., Skóra J., Stępień Ł., Szponar B., Otlewska A., Pielech-Przybylska K. (2015). Assessment of microbial contamination within working environments of different types of composting plants. J. Air Waste Manag. Assoc..

[27] Szulc J., Okrasa M., Majchrzycka K., Sulyok M., Nowak A., Szponar B., Górczyńska A., Ryngajłło M., Gutarowska B. (2022). Microbiological and toxicological hazard assessment in a waste sorting plant and proper respiratory protection. J. Environ. Manag..

[28] Brągoszewska E., Biedroń I., Hryb W. (2020). Microbiological Air Quality and Drug Resistance in Airborne Bacteria Isolated from a Waste Sorting Plant Located in Poland―A Case Study. Microorganisms.

[29] Nair A.T. (2021). Bioaerosols in the landfill environment: An overview of microbial diversity and potential health hazards. Aerobiologia.

[30] Kaźmierczuk M., Bojanowicz-Bablok A. (2014). Bioaerosol concentration in the air surrounding municipal solid waste landfill. Environ. Prot. Nat. Resour..

[31] Lis D.O., Ulfig K., Wlazło A., Pastuszka J.S. (2004). Microbial Air Quality in Offices at Municipal Landfills. J. Occup. Environ. Hyg..

[32] Breza-Boruta B. (2012). Bioaerosols of the municipal waste landfill site as a source of microbiological air pollution and health hazard. Ecol. Chem. Eng. A.

[33] Hrenovic J., Ivankovic T., Durn G., Dekic S., Kazazic S., Kisic I. (2019). Presence of carbapenem-resistant bacteria in soils affected by illegal waste dumps. Int. J. Environ. Health Res..

[34] Mor S., Ravindra K., Dahiya R.P., Chandra A. (2006). Leachate Characterization and Assessment of Groundwater Pollution Near Municipal Solid Waste Landfill Site. Environ. Monit. Assess..

[35] Aderemi A.O., Oriaku A.V., Adewumi G.A., Otitoloju A.A. (2011). Assessment of groundwater contamination by leachate near a municipal solid waste landfill. Afr. J. Environ. Sci. Technol..

[36] Chukwuma O.B., Rafatullah M., Tajarudin H.A., Ismail N. (2021). Bacterial Diversity and Community Structure of a Municipal Solid Waste Landfill: A Source of Lignocellulolytic Potential. Life.

[37] Liu S., Xi B.-D., Qiu Z.-P., He X.-S., Zhang H., Dang Q.-L., Zhao X.-Y., Li D. (2019). Succession and diversity of microbial communities in landfills with depths and ages and its association with dissolved organic matter and heavy metals. Sci. Total Environ..

[38] Krishnamurthi S., Chakrabarti T. (2013). Diversity of Bacteria and Archaea from a landfill in Chandigarh, India as revealed by culture-dependent and culture-independent molecular approaches. Syst. Appl. Microbiol..

[39] Song L., Wang Y., Zhao H., Long D.T. (2015). Composition of bacterial and archaeal communities during landfill refuse decomposition processes. Microbiol. Res..

[40] Liu Y., Zhang Y., Shi Y., Shen F., Yang Y., Wang M., Zhang G., Deng T., Lai S. (2021). Characterization of fungal aerosol in a landfill and an incineration plants in Guangzhou, Southern China: The link to potential impacts. Sci. Total Environ..

[41] Song L., Wang Y., Tang W., Lei Y. (2015). Bacterial community diversity in municipal waste landfill sites. Appl. Microbiol. Biotechnol..

[42] Winsley T.J., Snape I., McKinlay J., Stark J., van Dorst J.M., Ji M., Ferrari B.C., Siciliano S.D. (2014). The ecological controls on the prevalence of candidate division TM7 in polar regions. Front. Microbiol..

[43] Figueroa-Gonzalez P.A., Bornemann T.L.V., Adam P.S., Plewka J., Révész F., von Hagen C.A., Táncsics A., Probst A.J. (2020). Saccharibacteria as Organic Carbon Sinks in Hydrocarbon-Fueled Communities. Front. Microbiol..

[44] Loick N., Hobbs P.J., Hale M.D.C., Jones D.L. (2009). Bioremediation of Poly-Aromatic Hydrocarbon (PAH)-Contaminated Soil by Composting. Crit. Rev. Environ. Sci. Technol..

[45] Guo H., Chen C., Lee D.-J., Wang A., Ren N. (2013). Sulfur–nitrogen–carbon removal of Pseudomonas sp. C27 under sulfide stress. Enzyme Microb. Technol..

[46] Lalucat J., Bennasar A., Bosch R., García-Valdés E., Palleroni N.J. (2006). Biology of Pseudomonas stutzeri. Microbiol. Mol. Biol. Rev..

[47] Zeng J., Zhu Q., Wu Y., Lin X. (2016). Oxidation of polycyclic aromatic hydrocarbons using Bacillus subtilis CotA with high laccase activity and copper independence. Chemosphere.

[48] Desai C., Jain K., Madamwar D. (2008). Evaluation of In vitro Cr(VI) reduction potential in cytosolic extracts of three indigenous Bacillus sp. isolated from Cr(VI) polluted industrial landfill. Bioresour. Technol..

[49] Threedeach S., Chiemchaisri W., Watanabe T., Chiemchaisri C., Honda R., Yamamoto K. (2012). Antibiotic resistance of Escherichia coli in leachates from municipal solid waste landfills: Comparison between semi-aerobic and anaerobic operations. Bioresour. Technol..

[50] Huang L.-N., Zhou H., Zhu S., Qu L.-H. (2004). Phylogenetic diversity of bacteria in the leachate of a full-scale recirculating landfill. FEMS Microbiol. Ecol..

[51] Abiriga D., Jenkins A., Alfsnes K., Vestgarden L.S., Klempe H. (2021). Characterisation of the bacterial microbiota of a landfill-contaminated confined aquifer undergoing intrinsic remediation. Sci. Total Environ..

[52] Bookstaver M., Godfrin M.P., Bose A., Tripathi A. (2015). An insight into the growth of Alcanivorax borkumensis under different inoculation conditions. J. Pet. Sci. Eng..

[53] Kasai Y., Kishira H., Sasaki T., Syutsubo K., Watanabe K., Harayama S. (2002). Predominant growth of Alcanivorax strains in oil-contaminated and nutrient-supplemented sea water. Environ. Microbiol..

[54] Romero M., Avendaño-Herrera R., Magariños B., Cámara M., Otero A. (2010). Acylhomoserine lactone production and degradation by the fish pathogen Tenacibaculum maritimum, a member of the Cytophaga-Flavobacterium-Bacteroides (CFB) group. FEMS Microbiol. Lett..

[55] Mobasseri M., Hutchinson M.C., Afshar F.J., Pedram M. (2019). New evidence of nematode-endosymbiont bacteria coevolution based on one new and one known dagger nematode species of Xiphinema americanum-group (Nematoda, Longidoridae). PLoS ONE.

[56] Bhati T., Gupta R., Yadav N., Singh R., Fuloria A., Waziri A., Chatterjee S., Purty R. (2019). Assessment of Bioremediation Potential of Cellulosimicrobium sp. for Treatment of Multiple Heavy Metals. Microbiol. Biotechnol. Lett..

[57] Wu Y.-R., He J. (2015). Characterization of a xylanase-producing Cellvibrio mixtus strain J3-8 and its genome analysis. Sci. Rep..

[58] Sekiguchi T., Saika A., Nomura K., Watanabe T., Watanabe T., Fujimoto Y., Enoki M., Sato T., Kato C., Kanehiro H. (2011). Biodegradation of aliphatic polyesters soaked in deep seawaters and isolation of poly(ɛ-caprolactone)-degrading bacteria. Polym. Degrad. Stab..

[59] Delacuvellerie A., Cyriaque V., Gobert S., Benali S., Wattiez R. (2019). The plastisphere in marine ecosystem hosts potential specific microbial degraders including Alcanivorax borkumensis as a key player for the low-density polyethylene degradation. J. Hazard. Mater..

[60] Rivero M., Alonso J., Ramón M.F., Gonzales N., Pozo A., Marín I., Navascués A., Juanbeltz R. (2019). Infections due to Cellulosimicrobium species: Case report and literature review. BMC Infect. Dis..

[61] Doron S., Gorbach S.L. (2008). Bacterial Infections: Overview. International Encyclopedia of Public Health.

[62] Frączek K., Kozdrój J., Górny R.L., Cyprowski M., Gołofit-Szymczak M. (2017). Fungal air contamination in distinct sites within a municipal landfill area. Int. J. Environ. Sci. Technol..

[63] Money N.P. (2016). Fungal Diversity. The Fungi.

[64] Klocke B., Flath K., Miedaner T. (2013). Virulence phenotypes in powdery mildew (Blumeria graminis) populations and resistance genes in triticale (x Triticosecale). Eur. J. Plant Pathol..

[65] Crous P.W., Summerell B.A., Mostert L., Groenewald J.Z. (2008). Host specificity and speciation of Mycosphaerella and Teratosphaeria species associated with leaf spots of Proteaceae. Persoonia-Mol. Phylogeny Evol. Fungi.

[66] Kirk P., Cannon P., Minter D., Stalpers J. (2011). Dictionary of the Fungi.

[67] Redhead S.A., Vilgalys R., Moncalvo J., Johnson J., Hopple J.S. (2001). Coprinus Pers. and the disposition of Coprinus species sensu lato. Taxon.

[68] Kordalewska M., Jagielski T., Brillowska-Dąbrowska A. (2016). Rapid Assays for Specific Detection of Fungi of Scopulariopsis and Microascus Genera and Scopulariopsis brevicaulis Species. Mycopathologia.

[69] Gopal K., Kalaivani V., Anandan H. (2020). Pulmonary infection by Chrysosporium species in a preexisting tuberculous cavity. Int. J. Appl. Basic Med. Res..

[70] Spampinato C., Leonardi D. (2013). Candida Infections, Causes, Targets, and Resistance Mechanisms: Traditional and Alternative Antifungal Agents. Biomed Res. Int..

[71] Saunders C.W., Scheynius A., Heitman J. (2012). Malassezia Fungi Are Specialized to Live on Skin and Associated with Dandruff, Eczema, and Other Skin Diseases. PLoS Pathog..

[72] Skóra J., Sulyok M., Nowak A., Otlewska A., Gutarowska B. (2017). Toxinogenicity and cytotoxicity of Alternaria, Aspergillus and Penicillium moulds isolated from working environments. Int. J. Environ. Sci. Technol..

[73] Pitt J.I., Hocking A.D. (2009). Fungi and Food Spoilage.

[74] Peixoto M.S., de Oliveira Galvão M.F., Batistuzzo de Medeiros S.R. (2017). Cell death pathways of particulate matter toxicity. Chemosphere.

[75] Mutlu E.A., Engen P.A., Soberanes S., Urich D., Forsyth C.B., Nigdelioglu R., Chiarella S.E., Radigan K.A., Gonzalez A., Jakate S. (2011). Particulate matter air pollution causes oxidant-mediated increase in gut permeability in mice. Part. Fibre Toxicol..

[76] Jiang L., Dai H., Sun Q., Geng C., Yang Y., Wu T., Zhang X., Zhong L. (2011). Ambient particulate matter on DNA damage in HepG2 cells. Toxicol. Ind. Health.

[77] Al-Nimer M.S.M., Hameed H.G., Mahmood M.M. (2015). Antiproliferative effects of aspirin and diclofenac against the growth of cancer and fibroblast cells: In vitro comparative study. Saudi Pharm. J..

[78] Marinov L., Georgieva A., Voynikov Y., Toshkova R., Nikolova I., Malchev M. (2021). Cytotoxic and antiproliferative effects of the nonsteroidal anti-inflammatory drug diclofenac in human tumour cell lines. Biotechnol. Biotechnol. Equip..

[79] Zulkarnain N.N., Anuar N., Johari N.A., Sheikh Abdullah S.R., Othman A.R. (2020). Cytotoxicity evaluation of ketoprofen found in pharmaceutical wastewater on HEK 293 cell growth and metabolism. Environ. Toxicol. Pharmacol..

[80] Kwon J.-T., Kim H.-M., Kim P., Choi K. (2014). Didecyldimethylammonium chloride induces oxidative stress and inhibits cell growth in lung epithelial cells. Mol. Cell. Toxicol..

[81] Xu X., Lu J., Sheng H., Zhang L., Gan T., Zhang J., Xu Y., Zhu X., Yang J. (2020). Evaluation of the cytotoxic and genotoxic effects by melamine and cyanuric acid co-exposure in human embryonic kidney 293 cells. Braz. J. Med. Biol. Res..

[82] Melekoğlu A., Ekici H., Arat E., Karahan S. (2020). Evaluation of melamine and cyanuric acid cytotoxicity: An in vitro study on L929 fibroblasts and CHO cell line. Ankara Üniv. Vet. Fakültesi Derg..

[83] Xu M.-L., Gao Y., Wang X., Xiong J., Chen X., Zhao S., Ma S., Huang Y., Liu J. (2016). Effect of carbaryl on some biochemical changes in PC12 cells: The protective effect of soy isoflavone genistein, and daidzein, and their mixed solution. CyTA-J. Food.

[84] Saquib Q., Siddiqui M.A., Ansari S.M., Alwathnani H.A., Musarrat J., Al-Khedhairy A.A. (2021). Cytotoxicity and genotoxicity of methomyl, carbaryl, metalaxyl, and pendimethalin in human umbilical vein endothelial cells. J. Appl. Toxicol..

[85] IARC (International Agency for Research on Cancer) (2022). IARC Monographs on the Evaluation of Carcinogenic Risks to Humans: Agents Classified by the IARC Monographs, Volumes 1–131.

