# Supplementary material for: Assessment of Physicochemical, Microbiological and Toxicological Hazards at an Illegal Landfill in Central Poland
Source: Int J Environ Res Public Health. 2022 Apr 15;19(8):4826. doi: 10.3390/ijerph19084826 (PMC9027659; doi:10.3390/ijerph19084826)
Supplement: Supplementary file 1 [file ijerph-19-04826-s001.zip › Table S1.pdf]

**Table S1.** Microbiological contamination of the air in the tested landfill

| Sampling sites | Number of microorganisms, CFU m$^{-3}$ | | | | | | | |
|---|---|---|---|---|---|---|---|---|
| | Bacteria | Mannitol-positive Staphylococci | *Enterobacteriace* | Haemolytic Staphylococci | *Pseudomonas fluorescens* | Fungi | Xerophilic fungi | Actinomycetes |
| A | Śr:4.47x10$^{2}$ | Śr:1.67x10$^{1}$ | Śr:<1.0x10$^{1}$ | Śr:1.67x10$^{1}$ | Śr:<1.0x10$^{1}$ | Śr:1.80x10$^{2}$ | Śr:1.80x10$^{2}$ | Śr:1.00x10$^{1}$ |
| | OS:4.04x10$^{1}$ | OS:5.77x10$^{0}$ | OS:- | SD:5.77x10$^{0}$ | SD:- | SD:2.00x10$^{1}$ | SD:2.00x10$^{1}$ | SD:0.00x10$^{0}$ |
| B | M:1.83x10$^{2}$ | M:2.33x10$^{1}$ | M:<1.0x10$^{1}$ | M:2.33x10$^{1}$ | M:<1.0x10$^{1}$ | M:3.27x10$^{2}$ | M:3.27x10$^{2}$ | M:2.33x10$^{1}$ |
| | SD:1.68x10$^{2}$ | SD:1.15x10$^{1}$ | SD:- | SD:1.15x10$^{1}$ | SD:- | SD:5.77x10$^{0}$ | SD:5.77x10$^{0}$ | SD:5.77x10$^{0}$ |
| C | M:1.10x10$^{3}$ | M:1.40x10$^{2}$ | M:<1.0x10$^{1}$ | M:1.40x10$^{2}$ | M:<1.0x10$^{1}$ | M:4.73x10$^{2}$ | M:4.73x10$^{2}$ | M:4.67x10$^{1}$ |
| | SD:2.08x10$^{1}$ | SD:3.00x10$^{1}$ | SD:- | SD:3.00x10$^{1}$ | SD:- | SD:1.04x10$^{2}$ | SD:1.04x10$^{2}$ | SD:5.77x10$^{0}$ |
| D | M:2.77x10$^{2}$ | M:8.00x10$^{1}$ | M:<1.0x10$^{1}$ | M:8.00x10$^{1}$ | M:<1.0x10$^{1}$ | M:3.83x10$^{2}$ | M:3.83x10$^{2}$ | M:1.67x10$^{1}$ |
| | SD:5.77x10$^{0}$ | SD:6.24x10$^{1}$ | SD:- | SD:6.24x10$^{1}$ | SD:- | SD:1.53x10$^{1}$ | SD:1.53x10$^{1}$ | SD:5.77x10$^{0}$ |
| E | M:3.30x10$^{2}$ | M:3.67x10$^{1}$ | M:<1.0x10$^{1}$ | M:3.67x10$^{1}$ | M:<1.0x10$^{1}$ | M:1.77x10$^{2}$ | M:1.77x10$^{2}$ | M:1.33x10$^{1}$ |
| | SD:1.25x10$^{2}$ | SD:1.53x10$^{1}$ | SD:- | SD:1.53x10$^{1}$ | SD:- | SD:2.52x10$^{1}$ | SD:2.52x10$^{1}$ | SD:5.77x10$^{0}$ |
| F | M:2.20x10$^{2}$ | M:1.33x10$^{1}$ | M:<1.0x10$^{1}$ | M:1.33x10$^{1}$ | M:<1.0x10$^{1}$ | M:1.53x10$^{2}$ | M:1.53x10$^{2}$ | M:3.33x10$^{0}$ |
| | SD:1.73x10$^{1}$ | SD:5.77x10$^{0}$ | SD:- | SD:5.77x10$^{0}$ | SD:- | SD:3.06x10$^{1}$ | SD:3.06x10$^{1}$ | SD:5.77x10$^{0}$ |
| G | M:9.33x10$^{1}$ | M:6.67x10$^{0}$ | M:<1.0x10$^{1}$ | M:6.67x10$^{0}$ | M:<1.0x10$^{1}$ | M:1.17x10$^{2}$ | M:1.17x10$^{2}$ | M:0.00x10$^{0}$ |
| | SD:4.04x10$^{1}$ | SD:5.77x10$^{0}$ | SD:- | SD:5.77x10$^{0}$ | SD:- | SD:1.53x10$^{1}$ | SD:1.53x10$^{1}$ | SD:- |

M - mean; SD - standard deviation
