# Supplementary material for: Assessment of Physicochemical, Microbiological and Toxicological Hazards at an Illegal Landfill in Central Poland
Source: Int J Environ Res Public Health. 2022 Apr 15;19(8):4826. doi: 10.3390/ijerph19084826 (PMC9027659; doi:10.3390/ijerph19084826)
Supplement: Supplementary file 1 [file ijerph-19-04826-s001.zip › Table S2.pdf]

Table S2. Microbiological contamination of the leachetes in the tested landfill

No.	Number of microorganisms, CFU ml^{-1}								
	**Psychrophilic bacteria**	**Mesophilic bacteria**	**Fungi**	**Actinomycetes**	**Haemolytic Staphylococci**	***Enterobacteriaceae***	**Mannitol-positive Staphylococci**	***Pseudomonas fluorescens***	**Sulfate reducing anaerobes**
1	M:1.18x10^6	M:7.07x10^5	M:3.67x10^1	M:7.67x10^1	M:1.07x10^4	M:7.00x10^3	M:1.47x10^4	M:1.33x10^1	M:1.70x10^3
	SD:9.45x10^4	SD:1.89x10^5	SD:5.77x10^0	SD:5.77x10^0	SD:5.77x10^2	SD:2.00x10^3	SD:1.53x10^3	SD:5.77x10^0	SD:4.58x10^2
2	M:1.74x10^6	M:8.57x10^5	M:6.67x10^1	M:8.67x10^1	M:1.27x10^4	M:1.43x10^4	M:1.20x10^4	M:6.67x10^0	M:3.83x10^3
	SD:4.58x10^4	SD:2.08x10^4	v5.77x10^0	SD:5.77x10^0	SD:1.15x10^3	SD:4.04x10^3	SD:2.00x10^3	SD:5.77x10^0	SD:3.06x10^2
3	M:2.69x10^6	M:1.18x10^6	M:3.67x10^1	M:1.80x10^2	M:3.33x10^3	M:7.33x10^3	M:4.33x10^3	M:3.00x10^3	M:6.37x10^3
	SD:3.00x10^4	SD:2.75x10^5	SD:5.77x10^0	SD:3.00x10^1	SD:1.15x10^3	SD:1.53x10^3	SD:5.77x10^2	SD:1.00x10^3	SD:1.10x10^3
4	M:5.00x10^4	M:5.33x10^4	M:4.67x10^1	M:2.10x10^2	M:4.33x10^3	M:7.33x10^1	M:7.33x10^3	M:1.33x10^1	M:2.97x10^3
	SD:1.00x10^4	SD:3.51x10^4	SD:5.77x10^0	SD:3.00x10^1	SD:5.77x10^2	SD:5.77x10^0	SD:1.53x10^3	SD:5.77x10^0	SD:1.38x10^3
5	M:3.33x10^4	M:3.37x10^3	M:8.00x10^1	M:8.67x10^1	M:6.67x10^2	M:2.23x10^2	M:1.67x10^3	M:1.33x10^1	M:5.83x10^1
	SD:5.77x10^3	SD:8.08x10^2	SD:2.00x10^1	SD:3.06x10^1	SD:5.77x10^2	SD:6.51x10^1	SD:5.77x10^2	SD:5.77x10^0	SD:1.42x10^1

M - mean; SD - standard deviation
