# Supplementary material for: Assessment of Physicochemical, Microbiological and Toxicological Hazards at an Illegal Landfill in Central Poland
Source: Int J Environ Res Public Health. 2022 Apr 15;19(8):4826. doi: 10.3390/ijerph19084826 (PMC9027659; doi:10.3390/ijerph19084826)
Supplement: Supplementary file 1 [file ijerph-19-04826-s001.zip › Table S3.pdf]

Table S3. Microbiological contamination of the leachetes in the tested landfill

No.	Number of microorganisms, CFU g^{-1}								
	**Psychrophilic bacteria**	**Mesophilic bacteria**	**Fungi**	**Actinomycetes**	**Haemolytic Staphylococci**	***Enterobacteriaceae***	**Mannitol-positive Staphylococci**	***Pseudomonas fluorescens***	**Sulfate reducing anaerobes**
1	M:8.53x10^5	M:5.49x10^5	M:1.63x10^2	M:4.67x10^4	M:7.30x10^5	M:1.87x10^3	M:1.68x10^6	M:2.67x10^2	M:2.92x10^5
	SD:5.51x10^4	SD:1.39x10^5	SD:6.11x10^1	SD:2.08x10^4	SD:1.30x10^5	SD:6.51x10^2	SD:4.93x10^4	SD:1.15x10^2	SD:3.88x10^5
2	M:1.09x10^6	M:1.00x10^6	M:1.67x10^2	M:3.00x10^4	M:1.83x10^5	M:2.33x10^4	M:4.03x10^5	M:6.67x10^1	M:6.03x10^4
	SD:5.51x10^4	SD:1.40x10^5	SD:4.51x10^1	SD:1.00x10^4	SD:1.53x10^4	SD:5.60x10^3	SD:4.73x10^4	SD:5.77x10^1	SD:1.34x10^4
3	M:8.53x10^5	M:8.97x10^5	M:1.87x10^2	M:5.67x10^4	M:9.50x10^5	M:7.32x10^4	M:1.08x10^6	M:3.33x10^1	M:2.66x10^5
	SD:2.07x10^5	SD:2.08x10^4	SD:4.04x10^1	SD:1.53x10^4	SD:2.01x10^5	SD:2.98x10^4	SD:1.21x10^5	SD:5.77x10^1	SD:1.66x10^4
4	M:1.28x10^6	M:1.74x10^6	M:1.83x10^2	M:5.67x10^4	M:9.70x10^5	M:1.46x10^4	M:9.13x10^5	M:0.00x10^0	M:4.59x10^5
	SD:9.61x10^4	SD:5.57x10^4	SD:3.06x10^1	SD:3.06x10^4	SD:3.46x10^4	SD:1.34x10^4	SD:1.20x10^5	SD:-	SD:6.42x10^4
5	M:9.33x10^5	M:1.18x10^6	M:1.83x10^2	M:5.67x10^4	M:5.43x10^5	M:1.47x10^3	M:3.47x10^5	M:0.00x10^0	M:8.90x10^4
	SD:6.66x10^4	SD:4.04x10^4	SD:6.66x10^1	SD:3.06x10^4	SD:5.77x10^4	SD:5.03x10^2	SD:5.69x10^4	SD:-	SD:7.21x10^3

M - mean; SD - standard deviation
