# Supplementary material for: Assessment of Physicochemical, Microbiological and Toxicological Hazards at an Illegal Landfill in Central Poland
Source: Int J Environ Res Public Health. 2022 Apr 15;19(8):4826. doi: 10.3390/ijerph19084826 (PMC9027659; doi:10.3390/ijerph19084826)
Supplement: Supplementary file 1 [file ijerph-19-04826-s001.zip › Table S6.pdf]

Table S6 - Identification parameters of toxic compounds found in leachate and soil samples

TargetScreener database	sample code	Compound name	m/z meas.	RT [min]	Expected diagnostic Ions	Found diagnostic ions	Area of chromatographic peak of principal ion	S/N	Delta RT	Delta m/z [mDa]
PESTICIDES POS	SOIL1	BAC 14	332.3313	11.76	1	1	16714	14	0.16	0.11
WATER POS	SOIL1	Carbamazepine	237.1025	6.96	1	1	27916	22	0.02	0.28
WATER POS	SOIL2	Carbamazepine	237.1026	6.98	1	1	121610	78	0.04	0.37
PESTICIDES POS	SOIL1	Carbanilide	213.1022	7.5	1	1	104974	64	-0.03	-0.01
PESTICIDES POS	SOIL2	Carbanilide	213.1023	7.52	1	1	86459	52	-0.01	0.11
PESTICIDES POS	SOIL3	Carbanilide	213.1023	7.51	1	1	70506	56	-0.02	0.07
PESTICIDES POS	SOIL4	Carbanilide	213.1022	7.52	1	1	78295	50	-0.01	-0.05
PESTICIDES POS	SOIL5	Carbanilide	213.1022	7.53	1	1	80989	50	0	0.01
PESTICIDES POS	SOIL1	DDAC-C10	326.3781	12.11	1	1	277623	198	0.15	-0.06
PESTICIDES POS	SOIL2	DDAC-C10	326.3779	12.11	1	1	258770	162	0.15	-0.25
PESTICIDES POS	SOIL3	DDAC-C10	326.3782	12.11	1	1	293702	258	0.15	0.12
PESTICIDES POS	SOIL4	DDAC-C10	326.3782	12.11	1	1	240521	149	0.15	0.06
PESTICIDES POS	SOIL5	DDAC-C10	326.3781	12.11	1	1	239585	156	0.15	0
PESTICIDES POS	SOIL4	DEET (Diethyltoluamide)	192.1382	7.71	1	1	52667	35	0.06	-0.07
WATER POS	SOIL2	Diclofenac	296.0241	9.91	3	3	5763	4	0.24	0.15
PESTICIDES POS	SOIL1	Dimethylphthalate	195.0652	6.25	1	1	48606	36	0.09	-0.02
PESTICIDES POS	SOIL3	Dimethylphthalate	195.0651	6.25	1	1	45130	36	0.09	-0.1
PESTICIDES POS	SOIL4	Dimethylphthalate	195.065	6.25	1	1	34235	26	0.09	-0.2
PESTICIDES POS	SOIL5	Dimethylphthalate	195.0653	6.25	1	1	35281	25	0.09	0.09
PESTICIDES POS	SOIL5	Melamine	127.0725	1.54	2	2	30371	23	-0.42	-0.13
WATER POS	SOIL2	Prometryn	242.1434	9.84	2	2	795974	547	0.23	-0.04
PESTICIDES POS	SOIL2	Prometryn (Caparol)	242.1434	9.84	1	1	795974	547	0.23	-0.04
PESTICIDES POS	SOIL1	Pyrethrin I	329.2109	12.45	1	1	25449	19	-0.32	-0.26
PESTICIDES POS	SOIL2	Pyrethrin I	329.211	12.46	1	1	180707	139	-0.31	-0.13
PESTICIDES POS	SOIL3	Pyrethrin I	329.2113	12.46	1	1	363288	322	-0.31	0.16
PESTICIDES POS	SOIL4	Pyrethrin I	329.2111	12.46	1	1	64248	54	-0.31	-0.03
WATER POS	SOIL2	Terbutryn	242.1434	9.84	2	2	795974	547	0.07	-0.04
PESTICIDES POS	SOIL2	Terbutryn	242.1434	9.84	2	2	795974	547	0.07	-0.04
WATER POS	SOIL4	Terbutryn	242.1433	9.85	2	2	102488	75	0.08	-0.14
PESTICIDES POS	SOIL4	Terbutryn	242.1433	9.85	2	2	102488	75	0.08	-0.14
WATER POS	SOIL3	Triphenylphosphate	327.078	10.76	1	1	160018	161	0.06	-0.03
PESTICIDES POS	SOIL3	Triphenylphosphate	327.078	10.76	1	1	160018	161	0.06	-0.03
PESTICIDES POS	LEACHATE4	BAC 18	388.3944	13.54	2	2	76390	56	0.15	0.58
WATER POS	LEACHATE1	Carbamazepine	237.1026	6.98	1	1	25020	21	0.04	0.33
WATER POS	LEACHATE2	Carbamazepine	237.1025	6.97	1	1	48688	35	0.03	0.25

WATER POS	LEACH-ATE3	Carbamazepine	237.1031	6.96	1	1	26684	21	0.02	0.86
PESTICIDES POS	LEACH-ATE1	Carbanilide	213.1024	7.51	1	1	200496	145	-0.02	0.12
PESTICIDES POS	LEACH-ATE2	Carbanilide	213.1023	7.52	1	1	100233	64	-0.01	0.08
PESTICIDES POS	LEACH-ATE3	Carbanilide	213.1025	7.51	1	1	79164	55	-0.02	0.24
PESTICIDES POS	LEACH-ATE4	Carbanilide	213.1023	7.5	1	1	225682	129	-0.03	0.04
PESTICIDES POS	LEACH-ATE5	Carbanilide	213.1026	7.52	1	1	136234	82	-0.01	0.36
PESTICIDES POS	LEACH-ATE2	Carbaryl Frasoilm 145	145.0648	6.89	1	1	6653	8	0.03	0.04
PESTICIDES POS	LEACH-ATE1	DDAC-C10	326.3786	12.11	1	1	252665	195	0.15	0.45
PESTICIDES POS	LEACH-ATE2	DDAC-C10	326.3784	12.1	1	1	248940	172	0.14	0.23
PESTICIDES POS	LEACH-ATE4	DDAC-C10	326.3786	12.11	1	1	260334	164	0.15	0.5
PESTICIDES POS	LEACH-ATE5	DDAC-C10	326.3785	12.11	1	1	234785	152	0.15	0.35
PESTICIDES POS	LEACH-ATE1	DEET (Diethyltoluamide)	192.1384	7.72	1	1	114885	85	0.07	0.11
PESTICIDES POS	LEACH-ATE2	DEET (Diethyltoluamide)	192.1383	7.71	1	1	183576	125	0.06	-0.04
PESTICIDES POS	LEACH-ATE4	DEET (Diethyltoluamide)	192.1384	7.7	1	1	73039	47	0.05	0.12
PESTICIDES POS	LEACH-ATE5	DEET (Diethyltoluamide)	192.1386	7.71	1	1	46380	30	0.06	0.32
WATER POS	LEACH-ATE2	Diclofenac	296.0243	9.9	3	3	9285	10	0.23	0.32
PESTICIDES POS	LEACH-ATE1	Dimethylphthalate	195.0654	6.24	1	1	95469	63	0.08	0.21
PESTICIDES POS	LEACH-ATE2	Dimethylphthalate	195.0655	6.25	1	1	44786	30	0.09	0.27
PESTICIDES POS	LEACH-ATE3	Dimethylphthalate	195.0657	6.25	1	1	39387	26	0.09	0.5
PESTICIDES POS	LEACH-ATE4	Dimethylphthalate	195.0653	6.24	1	1	77572	44	0.08	0.08
PESTICIDES POS	LEACH-ATE5	Dimethylphthalate	195.0655	6.24	1	1	49358	35	0.08	0.32
WATER POS	LEACH-ATE1	Ketoprofen	255.102	7.95	2	2	24710	21	0.06	0.47
WATER POS	LEACH-ATE2	Ketoprofen	255.1015	7.95	2	2	108097	85	0.06	-0.02
WATER POS	LEACH-ATE3	Ketoprofen	255.1021	7.95	2	2	99564	72	0.06	0.56
PESTICIDES POS	LEACH-ATE1	Melamine	127.0728	1.86	2	2	1092839	617	-0.1	0.1
PESTICIDES POS	LEACH-ATE2	Melamine	127.0725	1.94	2	2	657599	461	-0.02	-0.17
PESTICIDES POS	LEACH-ATE3	Melamine	127.0726	1.98	2	2	561551	342	0.02	-0.06
WATER POS	LEACH-ATE4	Permethrin Peak 1	391.086	13.23	1	1	9078	8	-0.36	-0.25
PESTICIDES POS	LEACH-ATE4	Prallethrin Peak 1	301.1779	11.18	2	2	105325	84	0.12	-1.87
PESTICIDES POS	LEACH-ATE4	Prallethrin Peak 2	301.1779	11.18	2	2	105325	84	-0.11	-1.87
PESTICIDES POS	LEACH-ATE1	Triphenylphosphate	327.0781	10.77	1	1	17346	15	0.07	0.04
WATER POS	LEACH-ATE1	Triphenylphosphate	327.0781	10.77	1	1	17346	15	0.07	0.04
