# Supplementary figures and images for: Assessment of Physicochemical, Microbiological and Toxicological Hazards at an Illegal Landfill in Central Poland
Source: Int J Environ Res Public Health. 2022 Apr 15;19(8):4826. doi: 10.3390/ijerph19084826 (PMC9027659; doi:10.3390/ijerph19084826)

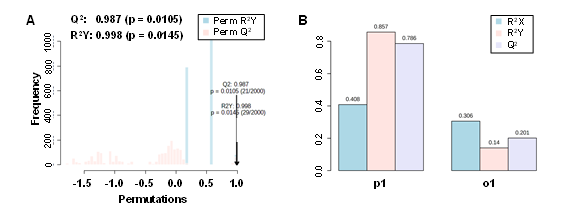

Supplement: Supplementary file 1 [file ijerph-19-04826-s001.zip › Figure S1.tif]

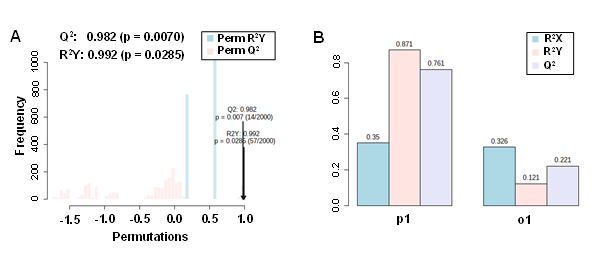

Supplement: Supplementary file 1 [file ijerph-19-04826-s001.zip › Figure S2.tif]
